# A multicentre study on grey matter morphometric biomarkers for classifying early schizophrenia and parkinson’s disease psychosis

**DOI:** 10.1038/s41531-023-00522-z

**Published:** 2023-06-08

**Authors:** Franziska Knolle, Shyam S. Arumugham, Roger A. Barker, Michael W. L. Chee, Azucena Justicia, Nitish Kamble, Jimmy Lee, Siwei Liu, Abhishek Lenka, Simon J. G. Lewis, Graham K. Murray, Pramod Kumar Pal, Jitender Saini, Jennifer Szeto, Ravi Yadav, Juan H. Zhou, Kathrin Koch

**Affiliations:** 1 Department of Diagnostic and Interventional Neuroradiology, School of Medicine, Technical University of Munich, Munich, Germany; 2 Department of Psychiatry, University of Cambridge, Cambridge, UK; 3 Department of Psychiatry, National Institute of Mental Health & Neurosciences (NIMHANS), Bengaluru, India; 4 Department of Clinical Neuroscience, University of Cambridge, Cambridge, UK; 5 Centre for Sleep and Cognition, Yong Loo Lin School of Medicine, National University of Singapore, Singapore, Singapore; 6 Centre for Translational MR Research, Yong Loo Lin School of Medicine, National University of Singapore, Singapore, Singapore; 7 IMIM (Hospital del Mar Medical Research Institute), Centro de Investigación Biomédica en Red de Salud Mental (CIBERSAM), Barcelona, Spain; 8 Department of Neurology, National Institute of Mental Health & Neurosciences (NIMHANS), Bengaluru, India; 9 Research Division, Institute of Mental Health, Singapore, Singapore; 10 Department of Psychosis, Institute of Mental Health, Singapore, Singapore; 11 Neuroscience and Mental Health, Lee Kong Chian School of Medicine, Nanyang Technological University, Singapore, Singapore; 12 Department of Neurology, Medstar Georgetown University School of Medicine, Washington, DC USA; 13 ForeFront Parkinson’s Disease Research Clinic, Brain and Mind Centre, School of Medical Sciences, University of Sydney, Camperdown, NSW Australia; 14 Cambridgeshire and Peterborough NHS Foundation Trust, Cambridge, UK

**Keywords:** Psychosis, Parkinson's disease

## Abstract

Psychotic symptoms occur in a majority of schizophrenia patients and in ~50% of all Parkinson’s disease (PD) patients. Altered grey matter (GM) structure within several brain areas and networks may contribute to their pathogenesis. Little is known, however, about transdiagnostic similarities when psychotic symptoms occur in different disorders, such as in schizophrenia and PD. The present study investigated a large, multicenter sample containing 722 participants: 146 patients with first episode psychosis, FEP; 106 individuals in at-risk mental state for developing psychosis, ARMS; 145 healthy controls matching FEP and ARMS, Con-Psy; 92 PD patients with psychotic symptoms, PDP; 145 PD patients without psychotic symptoms, PDN; 88 healthy controls matching PDN and PDP, Con-PD. We applied source-based morphometry in association with receiver operating curves (ROC) analyses to identify common GM structural covariance networks (SCN) and investigated their accuracy in identifying the different patient groups. We assessed group-specific homogeneity and variability across the different networks and potential associations with clinical symptoms. SCN-extracted GM values differed significantly between FEP and Con-Psy, PDP and Con-PD, PDN and Con-PD, as well as PDN and PDP, indicating significant overall grey matter reductions in PD and early schizophrenia. ROC analyses showed that SCN-based classification algorithms allow good classification (AUC ~0.80) of FEP and Con-Psy, and fair performance (AUC ~0.72) when differentiating PDP from Con-PD. Importantly, the best performance was found in partly the same networks, including the thalamus. Alterations within selected SCNs may be related to the presence of psychotic symptoms in both early schizophrenia and PD psychosis, indicating some commonality of underlying mechanisms. Furthermore, results provide evidence that GM volume within specific SCNs may serve as a biomarker for identifying FEP and PDP.

## Introduction

Psychotic symptoms, mostly occurring in the form of hallucinations or delusions, are highly debilitating; they may be treatment-resistant and often lead to poor functional outcomes^1^. They become manifest in different psychiatric and neurological disorders. In schizophrenia, psychotic symptoms constitute one of the core symptoms occurring in a majority of patients, mainly in the form of auditory and visual hallucinations^2,3^. Likewise, about 50% of all Parkinson’s disease (PD) patients suffer from psychotic symptoms, mainly in terms of visual and minor hallucinations^4^ that become more prominent during later stages of treated illness^5,6^. Across the different psychotic disorders, the pathogenesis of psychotic symptoms has been associated with alterations and altered interactions in a number of neurotransmitter systems, such as the dopaminergic, serotonergic, glutamatergic and cholinergic system. However, little is known about the commonalities of the substrates underlying psychotic symptoms in different disorders, such as schizophrenia and PD psychosis. Similarities in the neurobiology of those have been suggested, for example, in areas of prediction error processing^7,8^ and salience processing^9,10^, both linked to alterations in the dopaminergic systems^11,12^, as well as in mechanisms underlying auditory and visual hallucinations^13–15^. However, even less is known regarding disease-specific alterations in whole-brain grey matter (GM) pattern organisation. In psychosis, alterations in GM structure have been studied intensively, with mainly surface-based methods (SBM) and voxel-based morphometry (VBM)^16–20^.

Although meta-analyses have failed to arrive at any conclusive summary, they do suggest that alterations in several frontal and temporal regions, as well as the cingulate cortex and a number of subcortical areas, such as the hippocampus and the thalamus are among the most consistent findings^16,17,21^. These alterations seem to be present in help-seeking patients with an increased clinical risk of developing psychosis (i.e. individuals with an at-risk-mental state for developing psychosis, ARMS) and seem to progress during the course of the illness^22–24^.

Substantial efforts have been made to unravel GM structural alterations related to the presence of psychotic symptoms in PD^5,6,25–31^. A recent large-scale mega-analysis applied empirical Bayes harmonisation to identify structural alterations in PD patients with visual hallucinations compared to PD patients without visual hallucinations. After controlling for several influencing factors (i.e. age, gender, TIV, disease onset, medication, PD severity and cognition), they detected differences in cortical thickness and surface area in a wide-spread network comprising the primary visual cortex and its surrounding areas, and the hippocampus^31^. The authors concluded that their findings pointed to the involvement of the attentional control networks in the pathogenesis of PD visual hallucinations, supporting the attentional network hypothesis as proposed by Shine and colleagues^32^. Findings from a review by Lenka et al. (2015)^33^ suggested GM alterations in multiple regions of the brain, including, in addition to the primary visual cortex and hippocampus, frontoparietal regions, as well as the thalamus in PD patients with psychotic symptoms compared to those without. Those studies suggest that the GM alterations might be closely associated with the pathogenesis of psychotic symptoms in PD; however, they also illustrate that the overall picture is still heterogeneous, partly due to methodological differences between studies, but mostly because PD is regarded as a multi-systemic brain disease with diffuse alterations in multiple brain structures and functions.

In spite of all heterogeneity, there is a great overlap between those structures reported to be altered in psychosis patients and PD patients with psychotic symptoms, indicating that these alterations might represent a common underlying substrate of psychotic symptomatology. One of the major challenges when relating GM alterations in PD psychosis to those in schizophrenia is the difference in age of disease onset, with 60–80 years in PD^34^ and early 20s in psychosis patients^35^. Given the strong association between GM changes and age which, in turn, is closely related to illness duration, especially in elderly PD patients, age differences usually make it impossible to draw a clear conclusion on psychosis-related commonalities of structural alterations in these two disorders.

Based on these considerations, in the present study, we applied source-based morphometry (SBM) in association with receiver operating curves (ROC) analysis, to isolate common GM structural covariance networks (SCN) as a basis for potential diagnostic classification of the different patient groups while controlling for the highly relevant influence of age (i.e. by adding age as a covariate to the comparison of the different patient groups and having highly matched clinical and healthy control groups). More specifically, using this method, we aimed to identify SCN-related network characteristics that allow classification between ARMS, first episode psychosis (FEP) patients and PD patients with psychosis (PDP) versus respective controls. Identified networks may be closely related to psychotic symptoms. Importantly, networks showing similarly good classification performances for different patient groups would indicate potential commonality in underlying mechanisms. This way, we aim to explore whether similar networks occur within a disorder and across different stages (i.e. across FEP and ARMS), or across different disorders (i.e. across either FEP and PDP or ARMS and PDP). The latter comparison is especially interesting as it explores whether psychosis in PD corresponds to a manifest form of psychosis, as in comparison with FEP, to a subclinical form of psychosis, as in comparison with ARMS, or to neither of those.

However, since SCN identified by SBM have been shown to overlap with functional brain networks subserving behavioural and cognitive functions, they are gaining increasing importance as sensitive substrates for the right lingual gyrus, in the left lateral occipital gyrus and the right superior parietal lobe investigation of brain network organisation in neuropsychiatric diseases and are regarded as highly suitable for prediction or classification^36^. Nonetheless, to the best of our knowledge, there are only single studies investigating SCN in patients with psychosis^37–39^ and PD patients^40,41^, amongst these the above-mentioned large-scale mega-analysis^31^. In addition to the analyses mentioned before, they applied the structural covariance method to the cortical thickness and surface area in order to investigate grey matter network-level organisation in PD patients with vs. without visual hallucinations. They found, amongst others, significant differences in interregional surface area covariance and centrality in a wide-spread cortical network as well as more specific changes in cortical thickness in terms of greater betweenness centrality in PD patients with visual hallucinations compared to those without in the left and right lingual gyrus, in the left lateral occipital gyrus and the right superior parietal lobe.

Only one of these studies employed SCN-based classification in PD patients (without psychotic symptoms), and they reported an overall moderate SCN-related classification accuracy^40^. Thus, the present study aimed at investigating SCN-related GM alterations in patients with first episode psychosis, ARMS, as well as PD patients with and without psychotic symptoms to evaluate their suitability to identify psychosis-related characteristics considering age as a possible confounder. Finally, we aimed at exploring SCN-associated GM pattern organisation with regard to disease-specific characteristics in whole-brain GM patterns and their clinical relevance.

## Results

**Fig. 1:**
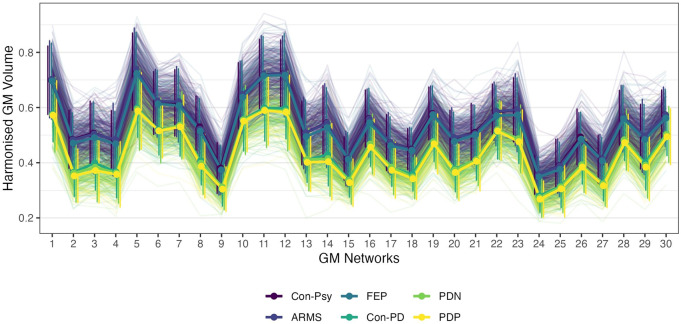
Mean GM values extracted from the 30 networks presented by the group. Line plot represents the mean and variance of harmonised grey matter values for each group and network. Dots and solid lines represent the group mean, and thin and vertical lines represent individuals and group distribution respectively.

**Fig. 2:**
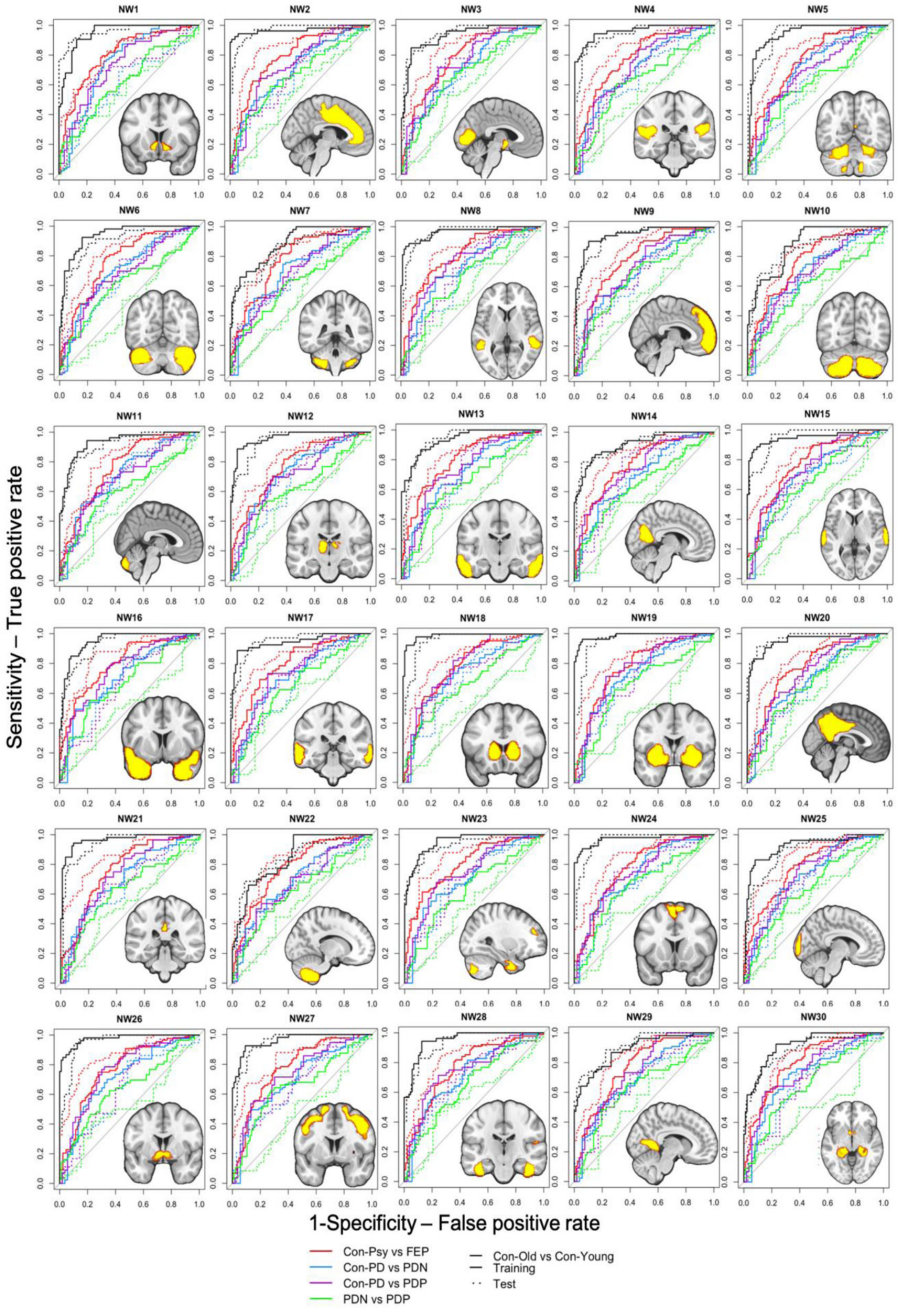
Classification performance of group differentiation. The 30 anatomically derived morphometric areas from the ICA networks thresholded at *z* = 3.5 and overlaid on the ROC curves for each group differentiation using harmonised grey matter values. Model training results are presented in solid lines, model evaluation in dotted lines. Black ROC: Con-Psy vs. Con-PD, red ROC: Con-Psy vs. FEP, blue ROC: Con-PD vs PDP; purple ROC: Con-PD vs. PDN.

Mean GM values extracted from each morphometric network and group are plotted in Fig. 1. The 30 morphometric networks are shown in Fig. 2 and their anatomical description as determined by the probability maps implemented in the JuBrain Anatomy toolbox^42^ can be found in the Supplementary Materials. The majority of morphometric networks showed a bilateral, mainly homotopic distribution. The 30 networks described clearly involve separate areas consisting of a large part of subcortical regions.

### Grey matter volume differences between groups

Results of the repeated-measures ANCOVA, with GM volume of the 30 networks as the within-subject factor, group as the between-subject factor and age, TIV, gender and scan site as covariates, showed a significant main effect of group (*F*(1, 712) = 10.99, *p* < 0.001), a significant main effect of network-related GM volume (*F*(8, 5750) = 44.18, *p* < 0.001), and significant interactions of network-related GM volume with age (*F*(8, 5750) = 12.50, *p* < 0.001), TIV (*F*(8, 5750) = 22.50, *p* < 0.001), gender (*F*(8, 5750) = 4.59, *p* < 0.001) and group (*F*(41, 5750) = 1.72, *p* < 0.003). The interaction with scan site was not significant (*F*(8, 5750) = 0.07, *p* < 1.0). All within-subject effects were Greenhouse-Geisser corrected due to a significant result in the Mauchly sphericity test. The repeated-measures ANCOVA comparing FEP vs. Con-Psy, Con-PD vs. PDN, Con-PD vs. PDP, FEP vs. PDN, ARM vs. PDN, PDN vs. PDP as well as young vs. elderly controls (Con-Psy vs. Con-PD) showed a significant main effect of group. Details of these results are presented in the Supplementary Materials.

**Fig. 3:**
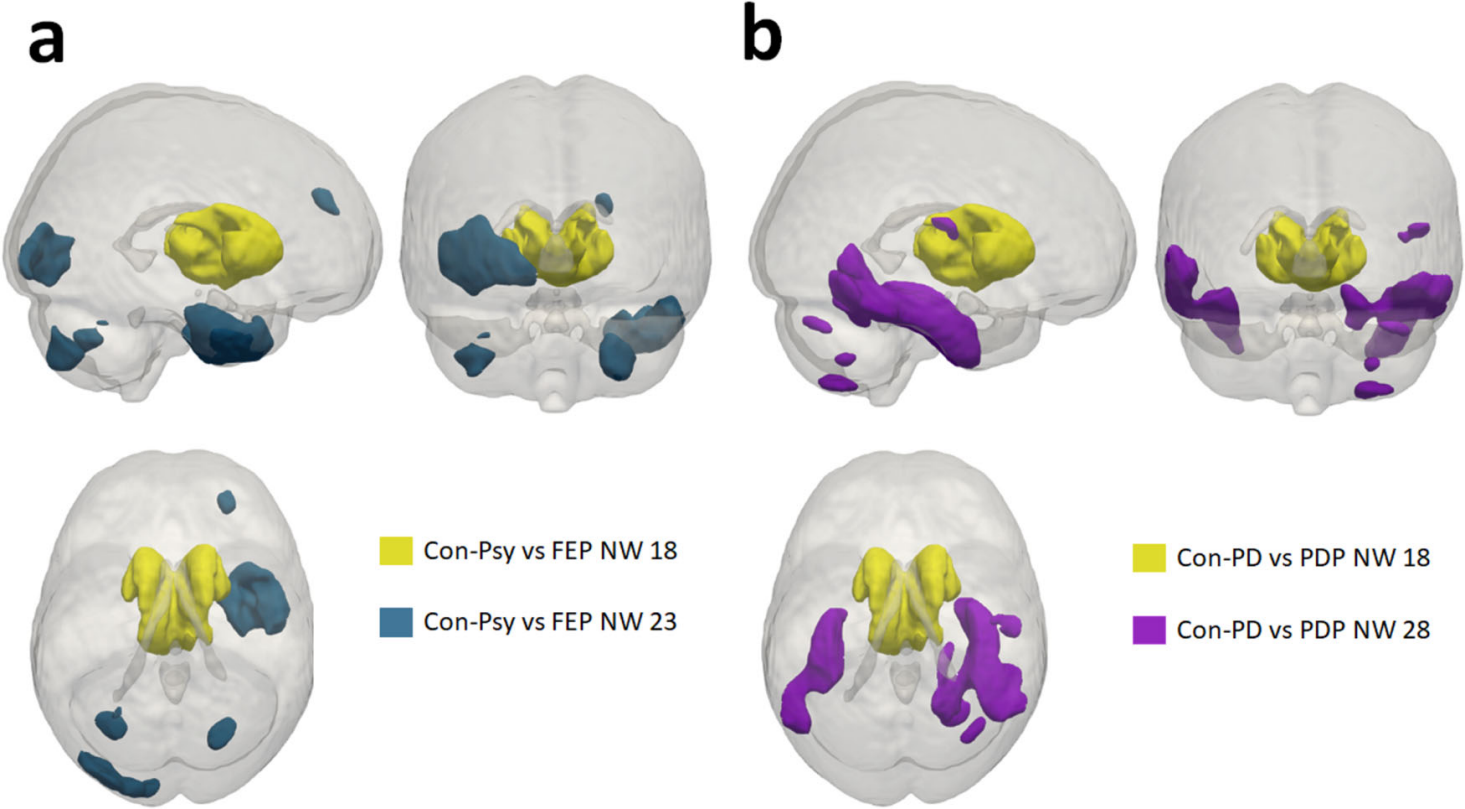
Best-classifying networks for FEP and PDP versus controls, with an overlap in the thalamus. **a** NW 18 and NW23 produced the best classification performance (AUC = 0.82 and AUC = 0.84) to discriminate FEP from Con-Psy; these NWs consist of the thalamus, the temporal fusiform cortex, the temporal pole, the occipital pole, the lateral occipital cortex, the cerebellum and the frontal pole. **b** NW 18 and 28, consisting of the thalamus, the parahippocampal gyrus, the temporal fusiform cortex, the cerebellum and Heschl’s gyrus, produced the best classification performance (AUC >0.73) to discriminate PDP from Con-PD.

The AUCs from the ROC analyses, representing the overall classification performance of each population-derived morphometric network to differentiate the different groups, are presented in Fig. 2 and the supplementary material Table 2. Classification performances differed depending on group comparison. The Con-Psy were differentiated from FEP with overall good performance in the training and the validation (AUCs average: 0.82 and 0.80, respectively). The Con-PD were differentiated from PDN with a fair performance (AUCs average: 0.74) in the training set and a poor performance (AUCs average: 0.64) in the validation. Similarly, Con-PD were differentiated from PDP with a fair performance in the training set (AUCs average: 0.76), and with a poor to fair performance (AUCs average: 0.69) in the validation. PDP and PND were classified poorly in the training (AUCs average: 0.63) and failed to classify in the validation set (AUCs average: 0.54). Classification of elderly (Con-PD) from young controls (Con-Psy), however, produced a mainly good to excellent performance in the training set (AUCs average: 0.94) and validation (AUCs average: 0.94; see ROC curves in Fig. 2). These results indicate that morphometric networks are suitable parameters to differentiate Con-Psy from FEP, as well as younger (Con-Psy) from elderly controls (Con-PD), and to a lesser degree also for the differentiation of Con-PD from PDP. The best-classifying networks for the comparison FEP vs. Con-Psy and PDP vs. Con-PD are presented in Fig. 3, showing an overlap in the NW 18, the thalamus.

Furthermore, we conducted control analyses removing one covariate at a time. The results are presented in Supplementary Tables 4–7. The impact of each covariate on the results is similar across all group comparisons indicating that group results are not dependent on covariates.

### Whole-brain grey matter pattern differences between groups

Assessment of GM pattern similarity (i.e. homogeneity) indicating how similar one’s whole-brain organisation is with every other individual of the respective group revealed a lower homogeneity in all patient groups (Con-Psy vs. FEP (*χ*2(59) = 403.25, *p* < 0.001); Con-Psy vs. ARMS (*χ*2(59) = 298.41, *p* < 0.001); Con-PD vs. PDN (*χ*2(59) = 454.34, *p* < 0.001); Con-PD vs. PDP (*χ*2(59) = 316.07, *p* < 0.001)).

### Differences in intra-network variability between groups

**Fig. 4:**
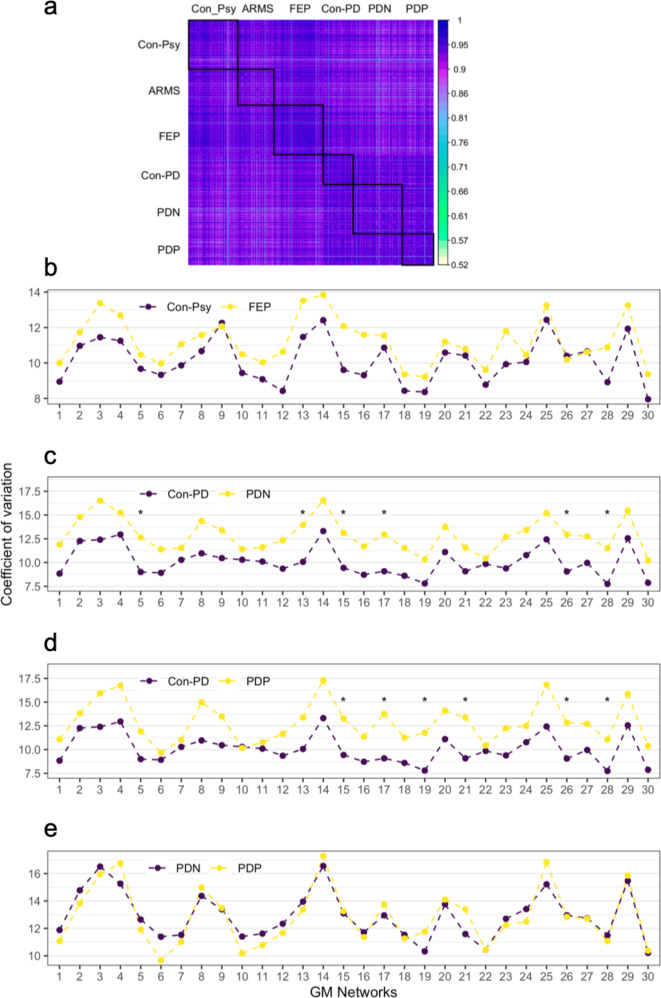
Homogeneity (i.e. inter-individual correlation in whole-brain grey matter patterns) and network-specific variability. **a** Homogeneity of GM volume per network and individual across all groups. The GM volume of each network for each individual is correlated with the GM volume of each NW of any other individual. Lighter colours indicate lower correlations. Black squares indicate groups. **b**–**e** Network-specific variability as assessed by the coefficient of variation for different group comparisons. **b** Con-Psy versus FEP; **c** Con-PD vs. PDN; **d** Con-PD vs. PDP, **e** PDN versus PDP. Group differences were investigated using the modified signed-likelihood ratio (MSLR) test; * significant at *p* < 0.002 corrected for multiple comparisons (i.e. 30 networks).

The MSLR test assessing differences in the coefficients of variation of GM volume between groups showed significant group effects between psychosis controls and FEP (*χ*2(1) = 9.13, *p* = 0.0025), and between Con-PD and PDN (*χ*2(1) = 10.97, *p* < 0.001), as well as highly significant group effects between Con-PD and PDP (*χ*2(1) = 15.21, *p* < 0.0001), indicating a higher variability in all patient groups across all networks (Fig. 4). We furthermore assessed differences for each network with each group comparison using a Bonferroni corrected threshold for multiple comparisons (*p* < 0.002), see Supplementary Table 3 for details. In summary, for the comparison between Con-Psy and FEP, as well as PDN and PDP, none of the significantly different networks survived multiple comparison corrections. For the comparison between Con-PD and PDN, we found significantly different, multiple comparisons corrected variability in NW5, NW13, NW15, NW17, NW26, and NW28; and between Con-PD and PDP in NW15, NW17, NW19, NW21, NW26 and NW28. All differences were based on an increased coefficient of variation (i.e. variability) in patients relative to healthy controls (see Fig. 4).

### Association with clinical scores

**Fig. 5:**
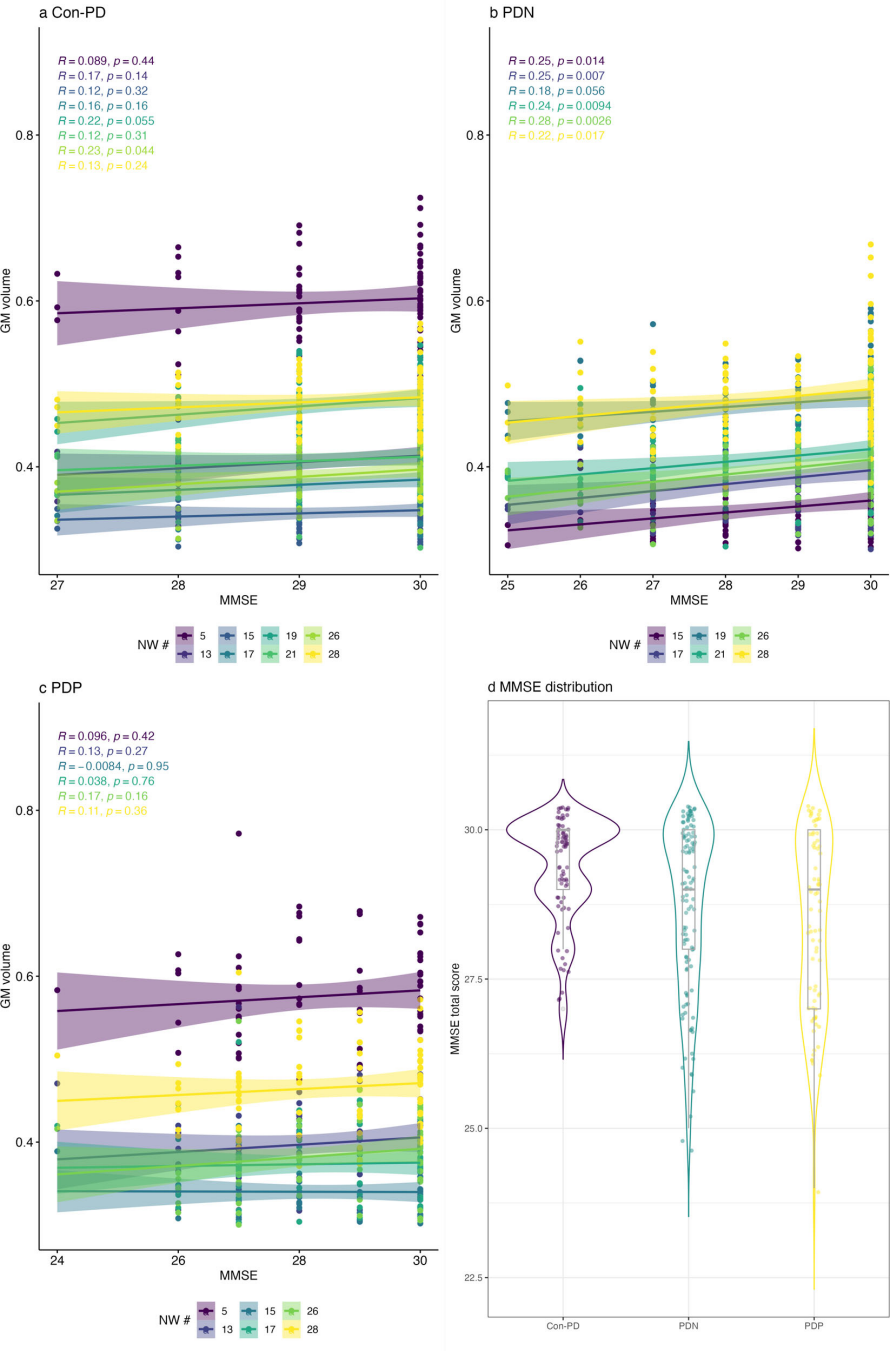
Correlation of cognitive scores with specific GM NWs which showed significantly different variability between controls and PD patients. A/B/C show correlations of GM NWs and MMSE in Con-PD (**a**), PDN (**b**) and PDP (**c**). While there are significant positive correlations in PDN and Con-Psy, indicating higher GM volume with less cognitive decline; there is no such correlation in PDP (**c**). **d** The violin plot shows the distribution of the MMSE scores across PDP, PDN and Con-PD, the box plots show individual scores, the median as a line and the mean as a dot. All analyses are controlled for multiple comparisons.

We did not find correlations between networks with increased variance and symptom scores in PDP. Interestingly however, in PDN, but not in PDP, we found a significant correlation between the GM volume of NW15, 17, 19, 21, 26, 28, and MMSE (*r* = 0.25, *p* = 0.014; *r* = 0.25, *p* = 0.007; *r* = 0.18, *p* = 0.056, *r* = 0.24, *p* = 0.0094, *r* = 0.28, *p* = 0.0026, *r* = 0.22, *p* = 0.017, respectively). The correlation implies lower GM volume with lower cognitive scores. In Con-PD, the correlation between the GM volume of NW19 and MMSE showed a trend towards significance and with NW26 and for the GM (*r* = 0.22, *p* = 0.055, *r* = 0.23, *p* = 0.044, respectively), indicating the same relationship as in PDN—greater GM volume with higher cognitive scores. Importantly, Con-PDs show a smaller range of cognitive scores, pointing towards less cognitive decline. All clinical associations are presented in Fig. 5.

## Discussion

This study aimed at investigating transdiagnostic GM differences and similarities between early schizophrenia and Parkinson’s Disease (PD) psychosis, in a unique sample that controls for age differences and disease stages, potentially shedding light on the development of psychotic symptoms in schizophrenia and PD. We present an SBM analysis, demonstrating widespread differences between patients and controls, with a general reduction of grey matter (GM) volume across the morphometric networks (NW), with a reduced inter-subject homogeneity, and increased intra-network variability in patients with both primary disorders. Importantly, we did not find differences in GM volume, homogeneity or variability between early schizophrenia and PD psychosis. Furthermore, data revealed that morphometric network-based classification algorithms show good performance when differentiating individuals with early schizophrenia (FEP) from healthy controls (Con-Psy), and a fair performance when differentiating individuals with PD psychosis (PDP) from healthy controls (Con-PD), with the best performance in a partly overlapping cluster containing the thalamus.

The ICA analysis identified 30 morphometric networks which clearly circumscribe cortical and subcortical areas using individual GM maps of all subjects. The structural covariance analysis revealed significant differences between patients and controls across both disorders—FEP vs. Con-Psy, PDN vs. Con-PD, PDP vs. Con-PD as well as PDN vs PDP. Interestingly, the comparison between psychosis-risk (ARMS) and Con-Psy, as well as comparisons between the patient groups (FEP vs. PDP, ARMS vs. FEP, ARMS vs. PDP), remained non-significant, potentially indicating similarities in the GM alterations across disease stages and disorders. Comparisons between ARMS vs. PDN, and FEP vs. PDN were conducted for completeness. As expected, those comparisons revealed significant results, most likely due to age-related alterations. GM alterations across the whole brain found in FEP compared to Con-Psy are in line with the literature reporting GM reductions across large areas of the brain^1,43^, including areas such as the anterior cingulate cortex (ACC), thalamus, insula and inferior frontal gyrus (IFG), superior temporal gyrus (STG), middle temporal gyrus (MTG), precuneus, and dorsolateral prefrontal cortex (DLPFC). They are in line with previous studies using SBM in patients with psychosis^38,39,44–48^. These studies reported decreased grey matter volume in mainly thalamic, frontal, temporal and parietal regions, although it should be noted that methodological details of the SBM approaches differed between the studies, and only two of those^38,45^ investigated patients with a first episode psychosis. Similarly, we found global, not NW-specific, reductions of GM volume across all NW in PDP and PDN compared to Con-PD. Psychosis, especially hallucinations in PD are associated with GM alterations in temporal and visual areas compared to non-psychotic PD patients^25^ and in the dorsal visual stream, the midbrain, cerebellar and limbic and paralimbic structures compared to healthy controls^28,31,33^. In this study, the structural covariance analysis likewise revealed significant overall GM differences between PDN and PDP, thus corroborating this earlier evidence for psychosis-related differences in GM volume in PD. Interestingly, there are no overall differences between FEP and PDP or PDN in the age-corrected GM NWs, potentially indicating similarities in structural changes^49,50^.

In our study, we did not find GM differences between ARMS and Con-Psy, despite several studies indicating such differences, especially in the insula, prefrontal and temporal brain regions^51–53^. The following considerations may explain the lack of findings in our sample. First of all, GM changes, especially in temporal and frontal areas, have been linked to symptom severity, particularly attenuated psychotic symptoms^54^, our sample of ARMS individuals is relatively mildly affected. Secondly, our sample combines European and Asian individuals (ratio 1:2); while all studies that report grey matter differences assess European, North-American or Australian participants^52,53,55,56^, a recent study reported no regional grey matter differences in an Asian sample^57^, discussing the lower prevalence of illegal drug use as a potential reason^48^. While this might provide a potential explanation, the general heterogeneity of this group might be equally likely. In the ARMS group, we did not differentiate between those who transition, or have an increased genetic risk, and those who remit. A systematic review^24^, however, showed that grey matter differences are more pronounced not only in high-risk individuals who transition into frank psychosis but also in those with a genetic risk compared to those who remitted, for whom it may also normalise. Thirdly, a recent meta-analysis in ARMS reported both increased and decreased GM volumes in different regions compared to healthy controls^58^. Given these inconsistent findings, it is possible that we did not find a significant overall (i.e., across all NW) group difference in GM volume in the current study for these reasons. As a proof of concept, we observed a strong decrease in GM volume, between young individuals and elderly individuals across all networks; as well as good to excellent classification performances^59^.

Is GM volume in NWs a suitable characteristic to identify individuals with early psychosis (i.e. FEP) or Parkinson’s psychosis (i.e. PDP)? Using logistic regression analysis, we found that morphometric NW patterns are suitable for the classification of FEP and Con-Psy with an overall fair performance (AUC >0.7). Networks that discriminated best (NW 2, 14, 16, 17, 23, all AUC = 0.84) included the cingulate gyrus, the frontal pole, the precuneus, the temporal pole, the parahippocampal gyrus, the orbitofrontal cortex, the lingual gyrus, the occipital fusiform gyrus, the lateral occipital cortex, the inferior temporal gyrus, the middle temporal gyrus, the cerebellum crus, the temporal fusiform cortex and the occipital pole. Those regions are highly relevant for psychopathology in psychosis, and structural alterations are well described in these areas^16,17,21,60–62^.

Importantly, several GM NWs also allowed fair classification performance when discriminating PDP from Con-PD (AUC >0.70). Brain regions of the best-classifying networks (AUC >0.72, NW 18, 28) include the thalamus, parahippocampal gyrus, temporal fusiform cortex, cerebellum and Heschl’s gyrus. Again, these regions have been discussed reliably in the literature as core structures for functional and structural alterations in PD with psychotic symptoms^25,27,28,31,33,63,64^.

Interestingly, the only study^31^ applying the structural covariance method to the cortical thickness and surface area in PD patients with vs. without visual hallucinations found, amongst others, significant differences in interregional surface area covariance in frontal and inferior-superior parietal regions, temporal fusiform areas, the lateral occipital gyrus, and insula as well as differences in betweenness centrality in PD patients with visual hallucinations compared to those without in the left and right lingual gyrus, in the left lateral occipital gyrus and the right superior parietal lobe. In the present study, classification between PD patients with visual hallucinations compared to those without yielded an overall poor performance, most probably since PD-associated changes might be prevailing and analysis was conducted at the network level such that the sensitivity to highly localised effects may have been reduced.

There is a strong overlap in fairly well-classifying regions between FEP and PDP, especially in the putamen, insula, hippocampus, parahippocampal gyrus precuneus, and thalamus. The presence of psychotic symptoms in this group of PD patients might introduce additional differentiating structural characteristics allowing for a better classification. Still, the specificity and sensitivity are reduced compared to the classification of early psychosis, which may result from a close association between age and illness duration in this particular group^65,66^. In a recent meta-analysis^67^ investigating progressive grey matter atrophy in individuals with PD, significant grey matter reductions were detected mainly in the caudate, putamen, n. accumbens, and amygdala. Our work shows that these regions overlap with areas affected and are used for the classification not only in PD with psychosis but also in early psychosis.

The classification of PD alone, without psychotic symptoms, was fair (max. AUC 0.76) in our sample. This is in line with a recent study by Lee and colleagues^40^, who were able to classify between PD patients and healthy controls with an accuracy of 0.75 in the validation sample, although this study did not differentiate between PD patients with and without psychotic symptoms. Despite the overlap in brain regions involved that seem to link to the presence of psychotic symptoms, it is not possible in this dataset to differentiate the contribution of specific psychotic symptoms, e.g. visual vs. auditory hallucinations. Our data, however, indicate that the thalamus classifies best in both PD patients with psychosis and psychosis patients (see Fig. 3). The thalamus is frequently being reported to show structural alterations in patients with psychosis^44,46,47^. The structure as a whole serves as a sensory and motor gateway to the cortex and provides a link between the cortex and the subcortical structures through cortico-striato-thalamic circuits. This way, the thalamus constitutes a filter for sensory and motor inputs to the cortex^68^. Structural alterations of the thalamus and its subnuclei are therefore discussed in light of a filter function failure resulting in both cognitive deficits as well as psychotic hallucinations. Likewise, in PD psychosis, the thalamus has been discussed as a key region linking several cortical networks associated with psychotic hallucinations^69^. The present results fortify these previous findings once more, suggesting a role of thalamic volume alterations in the psychopathology of psychosis and PD psychosis and indicate that this region might represent a common underlying substrate of psychotic symptomatology in both disorders.

Larger studies with distinguishable subgroups of symptom expression are needed to fully understand this potential target area.

As expected, when investigating correlations of an individual’s GM NW volumes to every other individual’s GM NW volumes, we found smaller homogeneity—or, in other terms, decreased inter-individual correlation in whole-brain grey matter patterns—in all patient groups compared to healthy controls. This decreased homogeneity may be linked to clinical symptomatology. These results are in line with findings in schizophrenia^70–72^ or Alzheimer’s Disease using a similar approach^59^. Both Parkinson’s disease and Psychosis are neurobiologically heterogeneous disorders^70,73^^.^ having multiple clinical subtypes, occurring with co-morbidities, and diverse representations across behaviour, genetics and brain morphometry. Relating to this, we, therefore, explored inter-individual GM volume variability; the variability was increased globally in FEP, PDP and PDN compared to their control groups. Additionally, we found specific NWs that showed increased variability. Despite overall increased variability in FEP across all NWs, differences in individual NWs did not survive multiple comparison correction.

In PDP compared to Con-PD variability was significantly greater in NW15, NW17, NW19, NW21, NW26 and NW28, comprising areas such as the n. accumbens, putamen, insula, posterior cingulate gyrus, temporal lobe, thalamus and cerebellum.

In contrast, PDN had increased variability in NW5, NW13, NW15, NW17, NW26, and NW28, including areas such as the cerebellum, n. accumbens, putamen, insula, thalamus and temporal lobe, which was, in turn, correlated with cognitive performance (i.e. MMSE score), indicating that reduced GM volume in PDN in these areas might be closely related to cognitive decline. Neither in Con-PD nor in PDP we found such an association.

Potential limitations need to be considered for this study. First, in a multi-cohort study, individuals from different studies are pooled together. Although we harmonised all grey matter volume values, parameters like scan-sites, imaging protocols and selection criteria might still introduce additional variance. In the ANCOVA and ROC analysis, we, therefore, additionally controlled for age, gender, scan site, and TIV to allow maximal comparability. However, a correction for age always entails removing the influence of disease (duration) to a limited degree, potentially reducing differences between patient and control groups. This constitutes a confound often present in PD and psychosis research. As each contributing study includes patients and controls assessed under identical circumstances, and each subject group consists of at least two different studies, intrinsic confounds are maximally controlled for. Nevertheless, we conducted logistic regression control analyses, removing one covariate at a time. As all covariates have a similar impact on the ROC results across all groups, this indicates that despite harmonisation there is still an effect of these covariates on grey matter volume, but that it is not specific to the group. Second, the clinical assessment varied across the different centres as well as across the different diseases. Therefore, no clinical score has consistently been used across all patient groups to assess psychotic symptoms in detail. We, however, made sure that each patient group, consisting of participants from multiple sites, had one identical clinical score, which unfortunately, was a sum score, combining different psychotic experiences. Therefore, the main disadvantage of this shortcoming is that symptom correlation cannot be studied in detail, and, thus, potential differences between the groups—such as a higher prevalence of visual hallucinations in PD, a higher percentage of auditory hallucinations in schizophrenia or the differentiation between illusions or hallucinations—cannot be considered. Furthermore, the lack of consistent clinical assessment also does not allow a separation of PD patients with visual illusions vs those with true visual hallucinations, which were found to differ substantially in functional connectivity but not grey matter volume. Third, as we are dealing with two different psychiatric diseases, schizophrenia and PD, with different medication strategies, for which a conversion into an equivalent dose is not possible, it is impossible to control for medication effects in the analysis. Therefore, the results could potentially be confounded by medication effects and/or duration of illness effects.

In conclusion, we were able to show that alterations in GM volume allow for the fair to the good classification of individuals with early psychosis and Parkinson’s psychosis. Furthermore, we found that there was reduced homogeneity and increased variability in patients compared to controls, potentially revealing those areas involved in the neurobiological processes underlying disease development.

Importantly, we found that the structure which classified best between FEP and controls, as well as PDP and controls was the thalamus, which is a region discussed as psychopathologically relevant for both psychosis as well as PD psychosis. Generally, a SCN approach may, therefore, not only be a powerful tool for the identification of individuals at risk for a disorder, but also for the understanding of transdiagnostic similarities and differences contributing to the development of certain symptoms.

## Methods

### Participants

In this study, we used a cross-sectional dataset to investigate early schizophrenia and Parkinson’s disease, combining imaging data from six original projects: the Early Psychosis Human Connectome Project (EP-HCP, https://www.humanconnectome.org/study/human-connectome-project-for-early-psychosis), an early schizophrenia dataset collected in Cambridge, UK^7,9^, an at-risk for psychosis dataset collected in Singapore^74^^.^ and three PD psychosis datasets, from Cambridge, UK^8,10^. Sydney, Australia^75^ and Bangalore, India^27^. The final dataset included 722 participants, consisting of: individuals with an at-risk mental state for developing psychosis (ARMS), showing sub-threshold positive and negative symptoms of schizophrenia; individuals with a first episode of psychosis (FEP), consisting of the first episode of schizophrenia and the first episode of schizoaffective disorder; healthy controls matching FEP and ARMS (Con-Psy); PD without psychosis (PDN); PD with psychosis (PDP); healthy controls matching PDN and PDP (Con-PD). Various clinical scores were recorded. Symptoms related to psychosis and schizophrenia were measured using the Comprehensive Assessment of At-Risk Mental States (CAARMS) in ARMS^76^ and the Positive and Negative Syndrome Scale (PANSS) in FEP^77^. In PD, the Hoehn and Yahr scale^78^ was used to assess the disease stage, and the Unified Parkinson’s Disease Rating Scale (UPDRS^79^) item 2 to assess psychotic symptoms and hallucinations. In PD, both the Mini-Mental State Examination (MMSE^80^) and the Montreal Cognitive Assessment (MoCA^81^) were used to assess cognitive decline. MoCA scores were converted to MMSE using a validated conversion table^82^. Demographic and clinical details, as well as corresponding statistics, are described in Table 1.

**Table 1:** Group demographics and clinical scores of the final sample.

Psychosis															
	Con-Psy				ARMS				FEP						comp.
	Cambridge	HCP	Singapore	Total	Cambridge	Singapore		Total	Cambridge	HCP			Total		KW-*X*^2^/P-*X*^2^, *X*^*2*^(df), p-value
*n*	49	57 (2x*)	39 (****)	145	32	74 (****)		106	23	123			146		n/a
Age, mean/SD (range)	23.18/3.37 (18–33)	24.88-4.08 (17–36)	22.51-3.96 (14–29)	23.67/3.93 (14–36)	21.44/3.29 (18–29)	21.46/3.43 (14–29)		21.45/3.38 (14–29)	22.78/5.18 (17–32)	22.84/3.86 (17–35)			22.83-4.07 (17–35)		22.15(2), <0.001
Sex, female	23	20	16	59	13	22		35	10	48			58		1.72(2), 0.4
CAARMS	n/a				16.56/7.57 (4–29)	16.05/7.47 (3–38)		16.21/7.47 (3–38)	-	-			-		n/a
PANSS	n/a				20.13/5.46 (14–38)	-		20.13/5.46 (14–38)	25.62/7.67 (16–51)	49.91/11.04 (30–78)			46.10/13.78 (16–78)		n/a
Medicated *****					0 (1 missing)	2		2	1 (1 missing)	90 (2 missing)			91		n/a

Parkinson’s Disease															
	Con-PD				PDP				PDN						
	Cambridge	Bangalore	Sydney	Total	Cambridge	Bangalore	Sydney	Total	Cambridge	Bangalore		Sydney	Total		
n	25 (***)	41	22	88	15	42	35 (**,***)	92	28	49 (*)		68 (2x*,****)	145		n/a
Age	62.2/5.92 (46-72)	55.42/5.23 (44-66)	67.73/8.17 (52-87)	60.42/8.03 (44-87)	61.93/7.47 (44-73)	58.43/8.54 (38-69)	65.83/7.02 (52-82)	61.82/8.45 (38-82)	63.07/9.58 (43-74)	57.87/6.84 (42-70)		66.84/8.66 (45-87)	63.08/9.14 (42-87)		7.27(2), 0.03
Sex, female	13	10	12	35	7	8	12	27	10	3		16	29		10.74 (2), 0.005
Hoehn&Yahr ******	n/a				1.71/0.91(1–3)	2.35/0.25(2–3)	2.09/0.46 (1–3)	2.16/0.53 (1–3)	1.48/0.90(1–5)	2.32/0.30(1.5–3)		2.11/0.76(1–5)	2.08/0.72(1–5)		n/a
UPDRS, Psychosis Scale	n/a				1.53/0.78 (1–3)	2.21/1.22 (1–4)	1.37/0.65 (1–3)	1.78/1.04 (1–4)	n/a						n/a
MMSE ******	29.5/079 (27–30)	29.15/0.91 (27–30)	29.74/0.56 (28–30)	29.37/0.84 (27–30	28.00/1.75 (24–30)	28.32/1.47 (26–30)	28.74/1.28 (7–30)	28.37/1.49 (24–30)	28.74/1.66 (25–30)		28.06/1.30 (25–30)	29.40/1.05 (27–30)		28.70/1.42 (25–30)	19.38 (2), <0.001
Medicated*******	n/a				15/0	42/0	34/0	91/0	28		49	63		140	n/a

Exclusion criteria from original data: * Missing files; ** faulty scan; *** listed multiple times; **** excluded during segmentation.

***** any current antipsychotic treatment, yes (N).

****** mean/SD (range).

******* Levodopa / antipsychotic, yes (N).

KW-*X*^*2*^ Kruskal–Wallis rank-sum test, P-*X*^*2*^ Pearson’s Chi-squared test.

Ethical approval was obtained from local ethical committees for each original studies: The studies were approved by the Cambridgeshire 3 National Health Service research ethics committee^8,10^; by the ethics review board of the Singaporean National Healthcare Group^74^; by the ethical committee of the University of Sydney^75^; and by the Institute Ethics Committee of NIMHANS, Bangalore^27^. Furthermore, freely available data was used from the Human Connectome Projects (https://www.humanconnectome.org/study/human-connectome-project-for-early-psychosis), for which ethical approval was waived by the Ethical Commission Board of the Technical University Munich. All subjects gave written informed consent in accordance with the Declaration of Helsinki.

### MRI acquisition, image preprocessing and independent component analysis

T1-weighted structural images were acquired for all individuals, at a field strength of 3T. The different MRI sequences are detailed in Supplementary Table 1. T1-weighted structural images were bias field corrected and segmented into grey matter, white matter, and CSF using Statistical Parametric Mapping (SPM12, http://www.fil.ion.ucl.ac.uk/spm/software/spm12/), running on MATLAB version 2018b. Diffeomorphic Anatomical Registration through Exponentiated Lie Algebra toolbox (DARTEL)^83^ was applied to grey matter images. This procedure created a sample-specific template representative of all 722 subjects by iterative alignment of all images. Subsequently, the template underwent non-linear registration with modulation for linear and non-linear deformations to the MNI-ICBM152 template. Each participant’s grey matter map was then registered to the group template and smoothed with an 8 mm^3^ isotropic Gaussian kernel.

### Independent component analysis

The independent component analysis (ICA) was conducted according to refs. ^59,84,85^. As a first step, all individually modulated and smoothed grey matter maps were concatenated to create a 4D file, which served as the basis for the independent component analysis (ICA). To ensure that only grey matter voxels were retained for the ICA, an absolute grey matter threshold of 0.1 was applied to all images. ICA was performed using the Multivariate Exploratory Linear Optimised Decomposition into Independent Components (MELODIC) method (http://fsl.fmrib.ox.ac.uk/fsl/fslwiki/MELODIC) as implemented in the FSL analysis package^86^ version 6.0. To derive data-driven population-based networks of grey matter covariance, the ICA was performed on all subjects (*n* = 722), thus identifying common spatial components based on the covariation of grey matter patterns across all participants. In line with previous work which employed similar methods, we chose 30 components^59,85,87^, which allows for the investigation of a relatively detailed organisation and represents one of the most frequent choices in resting state ICA analyses. To avoid spurious results, each of the 30 components or 30 morphometric networks was thresholded at *z* = 3.5 and binarized^59,85^. Finally, each participant’s grey matter volume was extracted from each of the 30 morphometric networks.

### Data harmonisation

We applied ComBat (Combating batch effects when combining Batches)^88^ to our processed structural grey matter data in order to reduce scanner effects across sites using a modified linear mixed effects model. In this model, we included all covariates of interest (i.e. age, gender and TIV). Thus, this harmonisation procedure reduces the effects of covariates linked to the different scanning sites, without altering the relationship between those and brain data. ComBat was implemented using R^89^. Harmonised data were used for all further analyses.

### Statistical analyses

#### Grey matter volume

To investigate group differences in harmonised GM volume across brain networks, we used repeated-measures ANCOVA with grey matter volume in the 30 networks as within-subjects factor and group as between-subjects factor. In post-hoc analyses, we performed comparisons between patient groups and their matched control groups (i.e. Con-Psy vs. FEP, Con-Psy vs. ARMS, Con-PD vs. PDN, Con-PD vs. PDP) and between all patient groups (i.e. FEP vs. ARMS, FEP vs. PDP, FEP vs. PDN, PDN vs. PDP, ARMS vs. PDN, ARMS vs. PDP). As proof of principle analysis, we conducted comparisons between young and elderly controls. Age, TIV, gender and scan site as covariates in all repeated-measures ANCOVAs, except for the comparison of elderly and young adults for which age was removed as a covariate.

We applied binary logistic regression models to examine the classification performance of the morphometric networks for those group comparisons showing a significant group difference in the ANCOVA. Previous studies showed that highly non-linear algorithms do not improve predictive performance when building a classifier based on image-derived brain data and for datasets in the size of the current one^90^. Therefore, a logistic regression model was used with harmonised GM volume as the predictor for group classification. The logistic regression models were controlled for age, gender, TIV and scan site for all group comparisons, except for young versus elderly healthy controls (Con-Psy vs. Con-PD), which excluded age as a covariate. We then performed receiver operating characteristic (ROC) analyses, and assessed the area under the curve (AUC) to evaluate the classification performance of each network. Logistic regressions, AUC and ROC analysis were computed using the glm and roc functions of the r-packages ‘stats’ and pROC^91^, respectively. We created a training and validation dataset by splitting the data using a 60:40 ratio. This ratio accounted for the different group sizes and allowed a minimum *N* = 50 in the training set, and furthermore avoided overfitting by allowing a minimum of *N* = 40 in the model evaluation. We generated the logistic regression model using the training data and tested the model using the validation data. AUC thresholds for classification were defined as follows: excellent = 0.90–1, good = 0.80–0.89, fair = 0.70–0.79, poor = 0.60–0.69 or fail = 0.50–0.59^92^.

#### Whole-brain grey matter pattern and intra-network variability

To investigate potential group differences in grey matter pattern similarity or homogeneity for those groups showing a significant group difference in the ANCOVAs, we correlated the grey matter volume in the 30 morphometric networks of each individual to the grey matter volume in the 30 brain networks of every other subject of the respective group^59,85^. Thus, homogeneity indicates the similarity of the whole-brain network profile from one subject with the whole-brain network profile of all other subjects in the group. To investigate whether groups differed in grey matter pattern similarity, we computed the Fligner-Killeen test of homogeneity of variances, using the fligner.test function of the r-package ‘stats’.

Finally, for those groups showing a significant group difference in the ANCOVAs, we investigated potential differences in the intra-network variability of grey matter volume between the groups by calculating the coefficient of variation (i.e. standard deviation divided by the mean of grey matter volume) in each of the 30 networks. The intra-network variability of grey matter volume between the groups in each of the 30 networks indicates the variability of the grey matter volume of each network between subjects. We calculated the modified signed-likelihood ratio (MSLR) test using the mslr function of the R-package ‘cvequality’ (https://cran.r-project.org/web/packages/cvequality/index.html) version 0.1.3^93^ with 100,000 simulations to test for significant differences in the coefficients of variation of grey matter volume between groups.

#### Correlations with clinical scores

We computed Pearson correlations between the grey matter volume of individual NWs (which showed significant differences in variability in group comparisons) and clinical scores, PANSS total and MMSE for FEP and PDP, respectively. We furthermore investigated associations between grey matter volume with the MDS-UPDRS Item 2 “Hallucination and Psychosis” score in PDP, which is a categorical score, using the Kruskal–Wallis test.

### Reporting summary

Further information on research design is available in the Nature Research Reporting Summary linked to this article.

## Supplementary information

Supplementary Material

Reporting Summary

## Data Availability

All data produced in the present study are available upon reasonable request to the authors (i.e. reasons for request should be explained). Given plausible reasons, there will be no restrictions on data sharing.
