# Supplementary material for: A multicentre study on grey matter morphometric biomarkers for classifying early schizophrenia and parkinson’s disease psychosis
Source: NPJ Parkinsons Dis. 2023 Jun 8;9:87. doi: 10.1038/s41531-023-00522-z (PMC10250419; doi:10.1038/s41531-023-00522-z)
Supplement: Supplementary file 1 — Supplementary Material [file 41531_2023_522_MOESM1_ESM.docx]

Supplementary materials

A multicentre study on grey matter morphometric biomarkers for classifying early schizophrenia and PD psychosis:

Knolle et al.

| Supplementary Table 1: Details for MPRAGE sequences by scanning location | | | | | |
| --- | --- | --- | --- | --- | --- |
| Study-location | RT (ms) | TE (ms) | Flip angle (degree) | FOV (mm) | Voxel size |
| Cambridge-  Psychosis | 2300 | 2.98 | 9 | 256x256 | 1x1x1 |
| EP-HCP | 2400 | 2.22 | 8 | 256x256 | 0.8x0.8x0.8 |
| Singapore-  Psychosis | 2300 | 2.98 | 8 | 256x256 | 1x1x1 |
| Sydney-PD | 7.2 | 2.7 | 12 | 256x256 | 1x1x1 |
| Cambridge-PD | 2300 | 2.98 | 9 | 256x256 | 1x1x1 |
| Bangalore-PD | 8.1 | 3.7 | 8 | 256X256 | 1x1x1 |

| Table 2. ROC diagnostics for classifications using training and test data sets per network and group comparison. | | | | | | | | | | | |
| --- | --- | --- | --- | --- | --- | --- | --- | --- | --- | --- | --- |
|  | Diagnostics  (in %) | Con-Psy vs. FEP | | Con-PD vs. PDN | | Con-PD vs. PDP | | PDN vs. PDP | | Con-Psy vs. Con-PD | |
| NW |  | Training | Test | Training | Test | Training | Test | Training | Test | Training | Test |
| 1 | Accuracy | 0.73 | 0.69 | 0.69 | 0.58 | 0.65 | 0.62 | 0.66 | 0.56 | 0.86 | 0.89 |
|  | Sensitivity | 0.66 | 0.69 | 0.70 | 0.57 | 0.66 | 0.46 | 0.94 | 0.83 | 0.87 | 0.90 |
|  | Specificity | 0.81 | 0.69 | 0.69 | 0.59 | 0.64 | 0.78 | 0.21 | 0.14 | 0.85 | 0.89 |
|  | AUC | 0.81 | 0.80* | 0.75 | 0.61 | 0.74 | 0.67 | 0.62 | 0.57 | 0.95 | 0.96* |
| 2 | Accuracy | 0.75 | 0.70 | 0.65 | 0.62 | 0.70 | 0.61 | 0.66 | 0.59 | 0.92 | 0.91 |
|  | Sensitivity | 0.71 | 0.67 | 0.62 | 0.66 | 0.72 | 0.51 | 0.86 | 0.83 | 0.90 | 0.91 |
|  | Specificity | 0.78 | 0.72 | 0.67 | 0.60 | 0.68 | 0.69 | 0.36 | 0.19 | 0.96 | 0.91 |
|  | AUC | 0.84 | 0.80* | 0.71 | 0.64 | 0.75 | 0.69 | 0.68 | 0.55 | 0.97 | 0.97* |
| 3 | Accuracy | 0.72 | 0.72 | 0.69 | 0.56 | 0.75 | 0.62 | 0.66 | 0.63 | 0.84 | 0.89 |
|  | Sensitivity | 0.66 | 0.78 | 0.70 | 0.60 | 0.79 | 0.54 | 0.90 | 0.84 | 0.84 | 0.88 |
|  | Specificity | 0.78 | 0.67 | 0.68 | 0.53 | 0.71 | 0.69 | 0.29 | 0.28 | 0.83 | 0.91 |
|  | AUC | 0.82 | 0.81* | 0.74 | 0.63 | 0.82 | 0.67 | 0.65 | 0.53 | 0.92 | 0.96* |
| 4 | Accuracy | 0.77 | 0.68 | 0.67 | 0.60 | 0.70 | 0.66 | 0.66 | 0.59 | 0.91 | 0.91 |
|  | Sensitivity | 0.72 | 0.69 | 0.66 | 0.63 | 0.75 | 0.54 | 0.95 | 0.84 | 0.91 | 0.91 |
|  | Specificity | 0.81 | 0.67 | 0.68 | 0.59 | 0.64 | 0.78 | 0.21 | 0.17 | 0.91 | 0.91 |
|  | AUC | 0.82 | 0.79~ | 0.72 | 0.64 | 0.75 | 0.69 | 0.61 | 0.50 | 0.96 | 0.96* |
| 5 | Accuracy | 0.72 | 0.70 | 0.68 | 0.59 | 0.70 | 0.62 | 0.69 | 0.55 | 0.83 | 0.94 |
|  | Sensitivity | 0.63 | 0.69 | 0.66 | 0.66 | 0.72 | 0.46 | 0.97 | 0.74 | 0.84 | 0.95 |
|  | Specificity | 0.81 | 0.71 | 0.69 | 0.55 | 0.68 | 0.78 | 0.27 | 0.25 | 0.81 | 0.91 |
|  | AUC | 0.81 | 0.80* | 0.74 | 0.62 | 0.74 | 0.68 | 0.64 | 0.55 | 0.94 | 0.96* |
| 6 | Accuracy | 0.73 | 0.71 | 0.66 | 0.58 | 0.64 | 0.61 | 0.65 | 0.61 | 0.81 | 0.87 |
|  | Sensitivity | 0.66 | 0.71 | 0.70 | 0.60 | 0.64 | 0.49 | 0.95 | 0.86 | 0.82 | 0.86 |
|  | Specificity | 0.81 | 0.71 | 0.64 | 0.57 | 0.64 | 0.72 | 0.18 | 0.19 | 0.81 | 0.89 |
|  | AUC | 0.81 | 0.79~ | 0.74 | 0.62 | 0.72 | 0.66 | 0.59 | 0.52 | 0.92 | 0.94* |
| 7 | Accuracy | 0.73 | 0.72 | 0.65 | 0.60 | 0.63 | 0.59 | 0.67 | 0.65 | 0.75 | 0.78 |
|  | Sensitivity | 0.64 | 0.74 | 0.66 | 0.60 | 0.66 | 0.46 | 0.95 | 0.93 | 0.75 | 0.76 |
|  | Specificity | 0.81 | 0.69 | 0.64 | 0.60 | 0.61 | 0.72 | 0.23 | 0.19 | 0.75 | 0.83 |
|  | AUC | 0.81 | 0.81* | 0.72 | 0.64 | 0.71 | 0.65 | 0.60 | 0.52 | 0.87 | 0.87* |
| 8 | Accuracy | 0.76 | 0.71 | 0.69 | 0.61 | 0.72 | 0.65 | 0.70 | 0.60 | 0.88 | 0.95 |
|  | Sensitivity | 0.74 | 0.71 | 0.66 | 0.71 | 0.77 | 0.60 | 0.94 | 0.78 | 0.89 | 0.97 |
|  | Specificity | 0.78 | 0.71 | 0.70 | 0.55 | 0.66 | 0.69 | 0.32 | 0.31 | 0.87 | 0.91 |
|  | AUC | 0.83 | 0.79~ | 0.72 | 0.65 | 0.77 | 0.70~ | 0.64 | 0.52 | 0.96 | 0.97* |
| 9 | Accuracy | 0.72 | 0.72 | 0.64 | 0.66 | 0.69 | 0.62 | 0.66 | 0.60 | 0.83 | 0.84 |
|  | Sensitivity | 0.67 | 0.71 | 0.60 | 0.71 | 0.68 | 0.54 | 0.86 | 0.86 | 0.83 | 0.83 |
|  | Specificity | 0.77 | 0.74 | 0.66 | 0.62 | 0.70 | 0.69 | 0.34 | 0.17 | 0.83 | 0.86 |
|  | AUC | 0.83 | 0.80* | 0.71 | 0.64 | 0.74 | 0.68 | 0.67 | 0.53 | 0.94 | 0.94* |
| 10 | Accuracy | 0.73 | 0.74 | 0.67 | 0.59 | 0.61 | 0.62 | 0.66 | 0.62 | 0.77 | 0.72 |
|  | Sensitivity | 0.64 | 0.74 | 0.70 | 0.60 | 0.66 | 0.51 | 0.94 | 0.84 | 0.77 | 0.74 |
|  | Specificity | 0.82 | 0.74 | 0.66 | 0.59 | 0.57 | 0.72 | 0.23 | 0.25 | 0.77 | 0.69 |
|  | AUC | 0.80 | 0.80* | 0.73 | 0.64 | 0.72 | 0.67 | 0.61 | 0.51 | 0.87 | 0.85* |
| 11 | Accuracy | 0.73 | 0.72 | 0.66 | 0.60 | 0.68 | 0.62 | 0.64 | 0.57 | 0.86 | 0.82 |
|  | Sensitivity | 0.66 | 0.71 | 0.66 | 0.60 | 0.68 | 0.51 | 0.92 | 0.81 | 0.87 | 0.81 |
|  | Specificity | 0.81 | 0.74 | 0.67 | 0.60 | 0.68 | 0.72 | 0.21 | 0.19 | 0.85 | 0.83 |
|  | AUC | 0.80 | 0.80* | 0.74 | 0.62 | 0.75 | 0.65 | 0.61 | 0.53 | 0.93 | 0.92* |
| 12 | Accuracy | 0.73 | 0.70 | 0.71 | 0.60 | 0.68 | 0.61 | 0.66 | 0.57 | 0.86 | 0.91 |
|  | Sensitivity | 0.66 | 0.71 | 0.72 | 0.66 | 0.70 | 0.46 | 0.90 | 0.79 | 0.84 | 0.95 |
|  | Specificity | 0.81 | 0.69 | 0.70 | 0.57 | 0.66 | 0.75 | 0.29 | 0.22 | 0.89 | 0.86 |
|  | AUC | 0.81 | 0.80* | 0.76 | 0.61 | 0.75 | 0.67 | 0.65 | 0.59 | 0.96 | 0.96* |
| 13 | Accuracy | 0.74 | 0.71 | 0.69 | 0.57 | 0.70 | 0.63 | 0.66 | 0.59 | 0.84 | 0.85 |
|  | Sensitivity | 0.67 | 0.71 | 0.68 | 0.63 | 0.70 | 0.51 | 0.94 | 0.86 | 0.83 | 0.84 |
|  | Specificity | 0.82 | 0.71 | 0.69 | 0.53 | 0.70 | 0.75 | 0.23 | 0.14 | 0.85 | 0.86 |
|  | AUC | 0.83 | 0.79~ | 0.74 | 0.62 | 0.76 | 0.68 | 0.60 | 0.57 | 0.93 | 0.93* |
| 14 | Accuracy | 0.77 | 0.71 | 0.66 | 0.59 | 0.75 | 0.61 | 0.67 | 0.56 | 0.79 | 0.84 |
|  | Sensitivity | 0.75 | 0.74 | 0.66 | 0.66 | 0.75 | 0.51 | 0.93 | 0.79 | 0.83 | 0.83 |
|  | Specificity | 0.80 | 0.67 | 0.67 | 0.55 | 0.75 | 0.69 | 0.27 | 0.19 | 0.74 | 0.86 |
|  | AUC | 0.84 | 0.79~ | 0.74 | 0.65 | 0.81 | 0.70~ | 0.65 | 0.50 | 0.90 | 0.93* |
| 15 | Accuracy | 0.74 | 0.70 | 0.68 | 0.63 | 0.70 | 0.65 | 0.67 | 0.53 | 0.89 | 0.89 |
|  | Sensitivity | 0.66 | 0.66 | 0.68 | 0.71 | 0.74 | 0.54 | 0.90 | 0.72 | 0.87 | 0.86 |
|  | Specificity | 0.82 | 0.74 | 0.68 | 0.59 | 0.66 | 0.75 | 0.32 | 0.22 | 0.92 | 0.94 |
|  | AUC | 0.81 | 0.79~ | 0.71 | 0.66 | 0.74 | 0.72~ | 0.66 | 0.54 | 0.96 | 0.95* |
| 16 | Accuracy | 0.79 | 0.72 | 0.69 | 0.57 | 0.72 | 0.63 | 0.66 | 0.63 | 0.86 | 0.88 |
|  | Sensitivity | 0.76 | 0.74 | 0.72 | 0.66 | 0.75 | 0.51 | 0.94 | 0.91 | 0.86 | 0.90 |
|  | Specificity | 0.82 | 0.71 | 0.67 | 0.52 | 0.70 | 0.75 | 0.21 | 0.17 | 0.85 | 0.86 |
|  | AUC | 0.84 | 0.81* | 0.74 | 0.63 | 0.76 | 0.72~ | 0.61 | 0.56 | 0.95 | 0.92* |
| 17 | Accuracy | 0.77 | 0.71 | 0.69 | 0.59 | 0.72 | 0.62 | 0.64 | 0.61 | 0.86 | 0.91 |
|  | Sensitivity | 0.74 | 0.74 | 0.70 | 0.69 | 0.75 | 0.49 | 0.92 | 0.83 | 0.86 | 0.90 |
|  | Specificity | 0.81 | 0.67 | 0.68 | 0.53 | 0.68 | 0.75 | 0.21 | 0.25 | 0.87 | 0.94 |
|  | AUC | 0.84 | 0.77~ | 0.73 | 0.62 | 0.77 | 0.68 | 0.63 | 0.55 | 0.94 | 0.95* |
| 18 | Accuracy | 0.73 | 0.72 | 0.72 | 0.61 | 0.74 | 0.66 | 0.69 | 0.55 | 0.90 | 0.95 |
|  | Sensitivity | 0.66 | 0.72 | 0.70 | 0.69 | 0.75 | 0.57 | 0.91 | 0.79 | 0.89 | 0.93 |
|  | Specificity | 0.81 | 0.72 | 0.74 | 0.57 | 0.73 | 0.75 | 0.34 | 0.17 | 0.92 | 0.97 |
|  | AUC | 0.81 | 0.82* | 0.76 | 0.66 | 0.79 | 0.74~ | 0.65 | 0.51 | 0.97 | 0.99* |
| 19 | Accuracy | 0.75 | 0.70 | 0.74 | 0.61 | 0.73 | 0.68 | 0.68 | 0.60 | 0.93 | 0.91 |
|  | Sensitivity | 0.69 | 0.71 | 0.74 | 0.69 | 0.77 | 0.60 | 0.92 | 0.86 | 0.92 | 0.90 |
|  | Specificity | 0.81 | 0.69 | 0.75 | 0.57 | 0.70 | 0.75 | 0.30 | 0.17 | 0.94 | 0.94 |
|  | AUC | 0.81 | 0.81* | 0.76 | 0.65 | 0.81 | 0.71~ | 0.63 | 0.52 | 0.97 | 0.98* |
| 20 | Accuracy | 0.75 | 0.72 | 0.66 | 0.59 | 0.71 | 0.56 | 0.67 | 0.57 | 0.89 | 0.86 |
|  | Sensitivity | 0.70 | 0.74 | 0.66 | 0.69 | 0.74 | 0.43 | 0.90 | 0.81 | 0.89 | 0.86 |
|  | Specificity | 0.80 | 0.69 | 0.66 | 0.53 | 0.68 | 0.69 | 0.32 | 0.19 | 0.89 | 0.86 |
|  | AUC | 0.83 | 0.78~ | 0.72 | 0.61 | 0.79 | 0.65 | 0.67 | 0.51 | 0.96 | 0.97* |
| 21 | Accuracy | 0.74 | 0.72 | 0.67 | 0.66 | 0.69 | 0.66 | 0.68 | 0.57 | 0.88 | 0.88 |
|  | Sensitivity | 0.67 | 0.74 | 0.68 | 0.74 | 0.75 | 0.54 | 0.94 | 0.84 | 0.87 | 0.86 |
|  | Specificity | 0.81 | 0.71 | 0.67 | 0.60 | 0.63 | 0.78 | 0.27 | 0.14 | 0.89 | 0.91 |
|  | AUC | 0.82 | 0.79~ | 0.72 | 0.64 | 0.76 | 0.68 | 0.63 | 0.55 | 0.96 | 0.97* |
| 22 | Accuracy | 0.73 | 0.72 | 0.66 | 0.60 | 0.62 | 0.58 | 0.66 | 0.63 | 0.79 | 0.72 |
|  | Sensitivity | 0.67 | 0.72 | 0.60 | 0.60 | 0.68 | 0.46 | 0.94 | 0.88 | 0.84 | 0.72 |
|  | Specificity | 0.78 | 0.71 | 0.69 | 0.60 | 0.57 | 0.69 | 0.23 | 0.22 | 0.72 | 0.71 |
|  | AUC | 0.82 | 0.80* | 0.71 | 0.64 | 0.72 | 0.64 | 0.62 | 0.52 | 0.86 | 0.83* |
| 23 | Accuracy | 0.75 | 0.75 | 0.70 | 0.57 | 0.74 | 0.61 | 0.68 | 0.62 | 0.86 | 0.84 |
|  | Sensitivity | 0.72 | 0.74 | 0.72 | 0.60 | 0.75 | 0.51 | 0.95 | 0.90 | 0.85 | 0.86 |
|  | Specificity | 0.77 | 0.76 | 0.69 | 0.55 | 0.73 | 0.69 | 0.25 | 0.17 | 0.87 | 0.80 |
|  | AUC | 0.84 | 0.82* | 0.75 | 0.63 | 0.79 | 0.69 | 0.60 | 0.55 | 0.95 | 0.94* |
| 24 | Accuracy | 0.74 | 0.71 | 0.69 | 0.58 | 0.73 | 0.61 | 0.64 | 0.54 | 0.89 | 0.91 |
|  | Sensitivity | 0.69 | 0.69 | 0.70 | 0.69 | 0.75 | 0.60 | 0.91 | 0.71 | 0.89 | 0.91 |
|  | Specificity | 0.80 | 0.72 | 0.69 | 0.52 | 0.71 | 0.61 | 0.23 | 0.28 | 0.91 | 0.91 |
|  | AUC | 0.82 | 0.78~ | 0.74 | 0.64 | 0.79 | 0.68 | 0.65 | 0.50 | 0.96 | 0.98* |
| 25 | Accuracy | 0.76 | 0.72 | 0.70 | 0.60 | 0.72 | 0.62 | 0.64 | 0.55 | 0.79 | 0.86 |
|  | Sensitivity | 0.69 | 0.76 | 0.70 | 0.66 | 0.74 | 0.54 | 0.93 | 0.79 | 0.80 | 0.93 |
|  | Specificity | 0.83 | 0.67 | 0.70 | 0.57 | 0.71 | 0.69 | 0.20 | 0.17 | 0.77 | 0.74 |
|  | AUC | 0.82 | 0.81* | 0.74 | 0.63 | 0.79 | 0.65 | 0.61 | 0.57 | 0.89 | 0.92* |
| 26 | Accuracy | 0.74 | 0.74 | 0.66 | 0.62 | 0.68 | 0.69 | 0.67 | 0.59 | 0.91 | 0.86 |
|  | Sensitivity | 0.68 | 0.74 | 0.64 | 0.69 | 0.68 | 0.60 | 0.92 | 0.83 | 0.89 | 0.86 |
|  | Specificity | 0.80 | 0.74 | 0.68 | 0.59 | 0.68 | 0.78 | 0.29 | 0.19 | 0.94 | 0.86 |
|  | AUC | 0.82 | 0.80* | 0.72 | 0.64 | 0.76 | 0.70~ | 0.65 | 0.57 | 0.98 | 0.95* |
| 27 | Accuracy | 0.75 | 0.72 | 0.66 | 0.61 | 0.64 | 0.68 | 0.68 | 0.59 | 0.86 | 0.89 |
|  | Sensitivity | 0.69 | 0.74 | 0.64 | 0.74 | 0.62 | 0.63 | 0.92 | 0.79 | 0.87 | 0.84 |
|  | Specificity | 0.81 | 0.69 | 0.67 | 0.53 | 0.66 | 0.72 | 0.30 | 0.25 | 0.85 | 0.97 |
|  | AUC | 0.83 | 0.78~ | 0.73 | 0.63 | 0.74 | 0.69 | 0.65 | 0.49 | 0.95 | 0.97* |
| 28 | Accuracy | 0.78 | 0.72 | 0.68 | 0.63 | 0.72 | 0.68 | 0.65 | 0.62 | 0.89 | 0.81 |
|  | Sensitivity | 0.72 | 0.72 | 0.66 | 0.74 | 0.74 | 0.57 | 0.92 | 0.88 | 0.90 | 0.83 |
|  | Specificity | 0.84 | 0.71 | 0.69 | 0.57 | 0.71 | 0.78 | 0.23 | 0.19 | 0.87 | 0.77 |
|  | AUC | 0.82 | 0.77~ | 0.75 | 0.64 | 0.76 | 0.73~ | 0.64 | 0.54 | 0.96 | 0.92* |
| 29 | Accuracy | 0.73 | 0.73 | 0.66 | 0.61 | 0.70 | 0.65 | 0.67 | 0.56 | 0.78 | 0.81 |
|  | Sensitivity | 0.66 | 0.72 | 0.70 | 0.69 | 0.72 | 0.54 | 0.89 | 0.81 | 0.77 | 0.83 |
|  | Specificity | 0.80 | 0.74 | 0.64 | 0.57 | 0.68 | 0.75 | 0.34 | 0.17 | 0.79 | 0.77 |
|  | AUC | 0.80 | 0.81* | 0.72 | 0.64 | 0.75 | 0.70~ | 0.66 | 0.56 | 0.89 | 0.91* |
| 30 | Accuracy | 0.74 | 0.74 | 0.71 | 0.61 | 0.72 | 0.65 | 0.70 | 0.62 | 0.81 | 0.75 |
|  | Sensitivity | 0.70 | 0.72 | 0.72 | 0.71 | 0.75 | 0.54 | 0.95 | 0.86 | 0.83 | 0.72 |
|  | Specificity | 0.78 | 0.76 | 0.71 | 0.55 | 0.70 | 0.75 | 0.30 | 0.22 | 0.77 | 0.80 |
|  | AUC | 0.83 | 0.80* | 0.74 | 0.67 | 0.76 | 0.70~ | 0.63 | 0.52 | 0.92 | 0.89* |
| *Note:* * Identifies networks with good classification performance or better in the validation (>=0.80); ~ identifies networks with fair classification performance in the validation (>=0.70). | | | | | | | | | | | |

| Supplementary Table 3: Modified signed likelihood ratio test (MSLRT) for equality of coefficients of variation | | | | | | | | |
| --- | --- | --- | --- | --- | --- | --- | --- | --- |
|  | Con-Psy vs. FEP | | Con-PD vs. PDN | | Con-PD vs. PDP | | PDN vs. PDP | |
| NW | MSLRT | P-value | MSLRT | P-value | MSLRT | P-value | MSLRT | p-value |
| 1 | 1.757 | 0.1850 | 8.851 | 0.003~ | 4.377 | 0.036 | 0.550 | 0.458 |
| 2 | 0.632 | 0.4265 | 3.575 | 0.059 | 1.227 | 0.268 | 0.491 | 0.484 |
| 3 | 3.339 | 0.0677 | 8.092 | 0.004 | 5.316 | 0.021 | 0.134 | 0.714 |
| 4 | 2.053 | 0.1519 | 2.776 | 0.096 | 5.565 | 0.018 | 0.900 | 0.343 |
| 5 | 0.879 | 0.3485 | 11.450 | 0.0007 | 6.736 | 0.010 | 0.395 | 0.530 |
| 6 | 0.617 | 0.4320 | 6.152 | 0.013 | 0.568 | 0.451 | 2.938 | 0.086 |
| 7 | 1.839 | 0.1751 | 1.416 | 0.234 | 0.419 | 0.517 | 0.233 | 0.629 |
| 8 | 0.951 | 0.3294 | 7.354 | 0.007 | 8.162 | 0.004 | 0.169 | 0.681 |
| 9 | 0.032 | 0.8571 | 6.184 | 0.013 | 5.462 | 0.019 | 0.003 | 0.958 |
| 10 | 1.603 | 0.2055 | 1.138 | 0.286 | 0.010 | 0.920 | 1.441 | 0.23 |
| 11 | 1.396 | 0.2374 | 2.080 | 0.149 | 0.367 | 0.545 | 0.646 | 0.422 |
| 12 | 7.584 | 0.0059 | 7.787 | 0.005 | 4.158 | 0.041 | 0.375 | 0.540 |
| 13 | 3.773 | 0.0521 | 10.656 | 0.0011 | 6.833 | 0.009 | 0.226 | 0.634 |
| 14 | 1.680 | 0.1949 | 4.859 | 0.028 | 5.697 | 0.017 | 0.181 | 0.670 |
| 15 | 7.247 | 0.0071 | 10.728 | 0.0011 | 9.756 | 0.002 | 0.011 | 0.917 |
| 16 | 6.679 | 0.0100 | 8.848 | 0.003 | 6.005 | 0.014 | 0.117 | 0.732 |
| 17 | 0.538 | 0.4632 | 12.570 | 0.0004 | 14.425 | 0.0002 | 0.372 | 0.542 |
| 18 | 1.548 | 0.2134 | 8.695 | 0.003~ | 6.148 | 0.013 | 0.069 | 0.792 |
| 19 | 1.331 | 0.2486 | 8.056 | 0.005 | 14.253 | 0.0002 | 1.785 | 0.181 |
| 20 | 0.459 | 0.4982 | 4.744 | 0.029 | 4.800 | 0.029 | 0.045 | 0.832 |
| 21 | 0.164 | 0.6853 | 6.083 | 0.014 | 12.631 | 0.0004 | 2.234 | 0.135 |
| 22 | 1.174 | 0.2785 | 0.370 | 0.543 | 0.294 | 0.588 | 0.0000 | 0.994 |
| 23 | 4.183 | 0.0408 | 9.193 | 0.002~ | 6.021 | 0.014 | 0.169 | 0.681 |
| 24 | 0.200 | 0.6547 | 4.912 | 0.027 | 1.886 | 0.170 | 0.561 | 0.454 |
| 25 | 0.564 | 0.4528 | 4.155 | 0.042 | 7.670 | 0.006 | 1.053 | 0.305 |
| 26 | 0.056 | 0.8137 | 12.806 | 0.0003 | 10.402 | 0.0013 | 0.0051 | 0.943 |
| 27 | 0.003 | 0.9577 | 6.165 | 0.013 | 5.023 | 0.025 | 0.0032 | 0.955 |
| 28 | 5.540 | 0.0186 | 15.399 | 0.0000 | 10.849 | 0.001 | 0.165 | 0.684 |
| 29 | 1.542 | 0.2143 | 4.462 | 0.035 | 4.590 | 0.032 | 0.058 | 0.809 |
| 30 | 3.733 | 0.0534 | 7.003 | 0.008 | 6.554 | 0.011 | 0.0219 | 0.882 |
| Group comparison across all NW | 9.16738 | 0.0024 | 10.936 | 0.0009 | 15.109 | 0.0001 | 1.0162 | 0.313 |
| *Note:* NW comparison corrected significance threshold: p<0.002; group comparison corrected significance threshold: p<0.012, bold=significant, *~=*trend (<0.0033) | | | | | | | | |

Anatomical description of ICA-derived grey matter networks

For each NW, we list the number of voxels, coordinates of the peak voxel, and brain region.

NW1:

1: 15422, 6, -54, -23, cerebellum VI

2: 451. -4, 4, -15, n. accumbens

3: 288, 12, 6, -12, n. accumbens, putamen

NW2:

1: 18791. 0. 14, 3, cingulate gyrus

2: 639, -24, 54, 8, frontal pole

3: 244, 15, -52, 6, precuneus

NW3:

1: 8596, 6, -75, 4, intracalcarine cortex, lingual gyrus

2: 518, -3, -8, -16, thalamus

NW4:

1: 6430. -39, -16, -12, central opercular cortex, insula

2: 4568, 40. -10. -15, parietal operculum cortex, insula

3: 269, -15, -73, -36, cerebellum crus II

NW5:

1: 6919, -7, -48, -27, cerebellum I-IV

2: 5664, 34, -42, -42, cerebellum VIIb, cerebellum VI

3: 239, 10. -66, -57, cerebellum VIIIa, cerebellum VIIIb

NW6:

1: 9979, 20. -60. -32, cerebellum crus I

2: 6259, -48, -69, -45, cerebellum crus I, cerebellum crus II

3: 179, -6, -4, -15, pallidum

4: 166, -4, -84, -2, lingual gyrus, intracalcarine cortex

NW7:

1: 7019, -15, -52, -48, cerebellum IX, cerebellum VIII

2: 6182, 26, -69, -60. cerebellum VIIIa, cerebellum VIIb

3: 694, -40. 42, -20. frontal pole

4: 320. -18, -60. -27, cerebellum VI

NW8:

1: 3814, 50. -10. -22, middle temporal gyrus

2: 2455, -50. -10. -22, middle temporal gyrus

3: 884, 51. -14, 32, postcentral gyrus

4: 781. -48, -16, 32, postcentral gyrus

5: 630. -28, -27, -24, parahippocampal gyrus

6: 478, 24, -33, -9, hippocampus

NW9:

1: 25689, 0. 54, 12, frontal pole, paracingulate gyrus

NW10:

1: 14233, 3, -74, -42, cerebellum vermis VIIIa, cerebellum VIIb

NW11:

1: 5684, 9, -74, -31. cerebellum crus I, cerebellum crus II

2: 610. -39, -68, -38, cerebellum crus I

3: 165, -33, -51. -56, cerebellum VIIIa

NW12:

1: 8747, -16, -60. -20. cerebellum VI

2: 875, -16, -26, -6, thalamus

3: 436, 14, -12, 4, thalamus

4: 340. 21. -70. -22, cerebellum VI, cerebellum crus I

NW13:

1: 6996, 41. 0. -33, inferior temporal gyrus, middle temporal gyrus

2: -48, 3, -48, inferior temporal gyrus, middle temporal gyrus, temporal pole

NW14:

1: 8803, 2, -64, 20. precuneus

NW15:

1: 5201. 68, -32, -12, middle temporal gyrus

2: 4514, -64, -33, -10. middle temporal gyrus

3: 567, 20. -4, -12, amygdala, n. accumbens

4: 420. -16, -4, -14, amygdala, n. accumbens

5: 255, 3, -60. -34, cerebellum vermis VIIIb, cerebellum vermis VIIIa

NW16:

1: 11821. -14, 0. -29, temporal pole, parahippocampal gyrus

2: 7202, 34, -6, -51. temporal pole, parahippocampal gyrus

3: 212, -26, 15, -20. orbitofrontal cortex

4: 115, -4, -72, 3, lingual gyrus

NW17:

1:13275, -38, -62, -8, occipital fusiform gyrus, lateral occipital cortex

2: 4314, 60. -45, -24, inferior temporal gyrus, middle temporal gyrus

3: 217, 51. 34, -14, frontal pole, orbitofrontal cortex

4: 204, 16, -75, -40. cerebellum crus II

NW18:

1: 12073, 2, 0. 3, thalamus

NW19:
1: 7507, -8, 4, -6, n. accumbens, putamen, insula

2: 7384, 33, 4, -22, n. accumbens, putamen, insula

NW20:

1: 11531. 2, -45, 38, posterior cingulate gyrus, precuneus

NW21:

1: 8446, 22, 21. -3, putamen, insula, orbitofrontal cortex

2: 6586, -33, 15, -27, insula, orbitofrontal cortex, temporal pole

3: 298, 0. -36, 24, posterior cingulate gyrus

NW22;

1: 11087, 2, -58, -46, cerebellum IX, cerebellum vermis IX

NW23:
1: 4388, 36, -20. -31. temporal fusiform cortex, temporal pole

2: 1782, -32, -92, -16, occipital pole, lateral occipital cortex

3: 703, 27, -81. -52, cerebellum crus II, cerebellum VIIb

4: 512, -33, -78, -51. cerebellum crus II, cerebellum VIIb

5: 197, 27, 50. 18, frontal pole

NW24:

1: 17918, 0. -30. 60. precentral gyrus, postcentral gyrus

2: 966, 27, -88, 27, lateral occipital cortex

NW25:

1: 7142, 8, -88, 6, intracalcarine cortex, occipital pole

2: 4539, -20. -96, -18, occipital pole

3: 112, 12, 22, -26, orbitofrontal cortex

NW26:

1: 15202, 0. 27, -14, subcallosal cortex

2: 1061. -9, -21. -6, thalamus

NW27:

1:16981. 21. 33, 35, superior frontal gyrus, frontal pole

2: 14607, -36, 40. -15, frontal pole

3: 612, -33, ,57, 36, lateral occipital cortex

4: 370. -26, 18, -24, orbitofrontal cortex

5: 205, 2, 0. 2, thalamus

NW28:

1: 6393, 18, 34, -18, parahippocampal gyrus

2: 3707, -34, -10. -46, temporal fusiform cortex

3: 371. 26, -69, -60. cerebellum VIIIa, cerebellum VIIb

4: 197, 48, -12, 8, Heschl’s gyrus

5: 159, 42, -69, -33, cerebellum crus I

NW29:

1: 3716, -3, -60. 6, lingual gyrus, precuneus

2: 3305, 21. -34, -22, parahippocampal gyrus

NW30:

1: 7078, -22, -10. -39, parahippocampal gyrus, hippocampus, amygdala

2: 6929, 14, -12, -22, parahippocampal gyrus, hippocampus, amygdala

3: 261. -4, 4, -15, n. accumbens

*Grey matter volume differences between groups - between-subject effects*

All within-subject effects were Greenhouse-Geisser corrected due to a significant result in the Mauchly sphericity test. The repeated-measures ANCOVA comparing FEP with Con-Psy showed a significant main effect of group (F(1. 285)=18.49, p<0.001) indicating there was a significantly smaller GM volume in patients compared to controls, a significant main effect of network-related GM volume (F(7, 2260)=14.21. p<0.001), and significant interactions of network-related GM volume with age (F(7, 2260)=2.33, p<0.02), and TIV (F(7, 2260)=8.24, p<0.001), and gender (F(7, 2260)=2.03, p<0.004). The interaction between network-related GM volume and scan site (F(7, 2260)=0.08, n.s.) was not significant. Again, all within-subject effects were Greenhouse-Geisser corrected due to a significant result in the Mauchly sphericity test. The repeated-measures ANCOVA comparing Con-PD with PDN showed a significant main effect of group (F(1. 227)=5.78, p<0.02) indicating a significantly smaller GM volume in patients compared to controls, a significant main effect of network-related GM volume (F(8, 1582)=20.41. p<0.001), and significant interactions of network-related GM volume with age (F(8, 1582)=12.86, p<0.001) and TIV (F(8, 1582)=9.30. p<0.001). The interaction between network-related GM volume and gender (F(8, 1582)=1.62, n.s.), scan site (F(8, 1582)=0.36, n.s.) and group (F(8, 1582)=1.53, n.s.) was not significant. Again, all within-subject effects were Greenhouse-Geisser corrected due to a significant result in the Mauchly sphericity test. The repeated-measures ANCOVA comparing Con-PD with PDP showed a significant main effect of group (F(1. 174)=12.86, p<0.001) with a significantly smaller GM volume in patients compared to controls, a significant main effect of network-related GM volume (F(7, 1191)=18.60. p<0.001), and significant interactions of network-related GM volume with age (F(7, 1191)=5.16, p<0.001), TIV (F(7, 1191)=3.16, p<0.003), gender (F(7, 1191)=3.92, p<0.001) and group (F(7, 1191)=2.09, p<0.04). The interaction between network-related GM volume and scan site was again not significant (F(7, 1191)=1.02, n.s.). All within-subject effects were Greenhouse-Geisser corrected due to a significant result in the Mauchly sphericity test. The repeated-measures ANCOVA comparing Con-Psy with Con-PD showed a significant main effect of group (F(1. 228)=345.7, p<0.001) indicating there was a significantly smaller GM volume in older compared to younger subjects, a significant main effect of network-related GM volume (F(8, 1739)=14.96, p<0.001), and significant interactions of network-related GM volume with TIV (F(8, 1739)=3.99, p<0.001), gender (F(8, 1739)=3.23, p<0.001) and group (F(8, 1739)=18.74, p<0.001) with multivariate post-hoc analyses showing significant group differences (i.e., smaller GM values in older compared to younger subjects) for all networks. The interaction between network-related GM volume and scan site was not significant (F(8, 1739)=0.43, n.s.). Again, all within-subject effects were Greenhouse-Geisser corrected due to a significant result in the Mauchly sphericity test. The repeated-measures ANCOVA comparing FEP with PDN showed a significant main effect of group (F(1. 285)=13.7, p<0.001) indicating there was a significantly smaller GM volume in PDN compared to FEP patients, a significant main effect of network-related GM volume (F(8, 2192)=20.96, p<0.001), and significant interactions of network-related GM volume with TIV (F(8, 2192)=13.57, p<0.001) and group (F(8, 2192)=3.69, p<0.001) with multivariate post-hoc analyses showing significant group differences (i.e., smaller GM values in PDN compared to FEP) for all networks. The interaction between network-related GM volume and scan site (F(8, 2192)=0.71. n.s.) as well as gender was not significant (F(8, 2192)=1.65, n.s.). Again, all within-subject effects were Greenhouse-Geisser corrected due to a significant result in the Mauchly sphericity test. The repeated-measures ANCOVA comparing ARMS with PDN showed a trend significant effect of group (F(1. 245)=3.25, p<0.07) indicating a smaller GM volume in PDN compared to ARMS patients, a significant main effect of network-related GM volume (F(8, 2032)=12.79, p<0.001), and significant interactions of network-related GM volume with TIV (F(8, 2032)=11.28, p<0.001) and gender (F(8, 2032)=2.36, p<0.01). The interaction between network-related GM volume and scan site was not significant (F(8, 2032)=0.36, n.s.). Again, all within-subject effects were Greenhouse-Geisser corrected due to a significant result in the Mauchly sphericity test. The repeated-measures ANCOVA comparing PDP with PDN showed a significant main effect of group (F(1. 231)=4.64, p<0.03) indicating a significantly smaller GM volume in PDP compared to PDN, a significant main effect of network-related GM volume (F(7, 1742)=20.86, p<0.001), and significant interactions of network-related GM volume with age (F(7, 1742)=11.89, p<0.001) and TIV (F(7, 1742)=8.92, p<0.001). The interaction between network-related GM volume and gender (F(7, 1742)=1.28, n.s.), scan site (F(7, 1742)=0.53, n.s.) and group (F(7, 1742)=0.81. n.s.) was not significant. Again, all within-subject effects were Greenhouse-Geisser corrected due to a significant result in the Mauchly sphericity test.

All other group comparisons did not reveal any significant results (FEP vs. PDP, FEP vs. ARMS, PDP vs. ARMS, ARMS vs. Con-Psy).

*Control analyses reducing the the ROC model by one covariate at a time*

| Supplementary Table 4. ROC diagnostics without age for classifications using training and test data sets per network and group comparison. | | | | | | | | | | | |
| --- | --- | --- | --- | --- | --- | --- | --- | --- | --- | --- | --- |
|  | Diagnostics  (in %) | Con-Psy vs. FEP | | Con-PD vs. PDN | | Con-PD vs. PDP | | PDP vs. PDN | | Con-Psy vs. Con-PD | |
| NW |  | Training | Test | Training | Test | Training | Test | Training | Test | Training | Test |
| 1 | Accuracy | 0.72 | 0.73 | 0.69 | 0.56 | 0.63 | 0.70 | 0.62 | 0.64 | 0.87 | 0.88 |
|  | Sensitivity | 0.60 | 0.62 | 0.70 | 0.51 | 0.66 | 0.74 | 1.00 | 1.00 | 0.89 | 0.83 |
|  | Specificity | 0.84 | 0.84 | 0.69 | 0.59 | 0.61 | 0.67 | 0.02 | 0.06 | 0.85 | 0.97 |
|  | AUC | 0.77 | 0.73 | 0.76 | 0.60 | 0.67 | 0.75 | 0.57 | 0.55 | 0.95 | 0.96 |
| 2 | Accuracy | 0.72 | 0.73 | 0.70 | 0.55 | 0.61 | 0.69 | 0.62 | 0.63 | 0.91 | 0.91 |
|  | Sensitivity | 0.60 | 0.62 | 0.77 | 0.46 | 0.64 | 0.74 | 0.99 | 1.00 | 0.92 | 0.86 |
|  | Specificity | 0.84 | 0.84 | 0.66 | 0.60 | 0.57 | 0.64 | 0.04 | 0.03 | 0.91 | 1.00 |
|  | AUC | 0.78 | 0.77 | 0.76 | 0.58 | 0.68 | 0.77 | 0.57 | 0.69 | 0.96 | 0.98 |
| 3 | Accuracy | 0.73 | 0.72 | 0.71 | 0.54 | 0.63 | 0.73 | 0.62 | 0.63 | 0.86 | 0.78 |
|  | Sensitivity | 0.62 | 0.62 | 0.75 | 0.51 | 0.68 | 0.83 | 0.99 | 1.00 | 0.86 | 0.76 |
|  | Specificity | 0.83 | 0.81 | 0.68 | 0.55 | 0.59 | 0.64 | 0.04 | 0.03 | 0.87 | 0.83 |
|  | AUC | 0.79 | 0.79 | 0.77 | 0.60 | 0.70 | 0.82 | 0.57 | 0.69 | 0.95 | 0.90 |
| 4 | Accuracy | 0.71 | 0.73 | 0.74 | 0.54 | 0.62 | 0.69 | 0.59 | 0.62 | 0.91 | 0.86 |
|  | Sensitivity | 0.60 | 0.62 | 0.75 | 0.49 | 0.68 | 0.77 | 0.98 | 1.00 | 0.91 | 0.83 |
|  | Specificity | 0.83 | 0.84 | 0.72 | 0.57 | 0.57 | 0.61 | 0.00 | 0.00 | 0.91 | 0.91 |
|  | AUC | 0.78 | 0.78 | 0.77 | 0.58 | 0.69 | 0.76 | 0.55 | 0.59 | 0.96 | 0.95 |
| 5 | Accuracy | 0.72 | 0.73 | 0.70 | 0.53 | 0.65 | 0.65 | 0.61 | 0.62 | 0.86 | 0.86 |
|  | Sensitivity | 0.60 | 0.62 | 0.70 | 0.46 | 0.70 | 0.74 | 0.99 | 1.00 | 0.86 | 0.81 |
|  | Specificity | 0.84 | 0.84 | 0.70 | 0.57 | 0.61 | 0.56 | 0.02 | 0.00 | 0.85 | 0.94 |
|  | AUC | 0.76 | 0.73 | 0.76 | 0.61 | 0.68 | 0.74 | 0.56 | 0.55 | 0.95 | 0.95 |
| 6 | Accuracy | 0.72 | 0.73 | 0.71 | 0.57 | 0.64 | 0.69 | 0.61 | 0.62 | 0.85 | 0.83 |
|  | Sensitivity | 0.60 | 0.62 | 0.74 | 0.51 | 0.68 | 0.74 | 0.97 | 0.97 | 0.85 | 0.76 |
|  | Specificity | 0.84 | 0.84 | 0.69 | 0.60 | 0.61 | 0.64 | 0.05 | 0.06 | 0.85 | 0.94 |
|  | AUC | 0.76 | 0.73 | 0.76 | 0.59 | 0.65 | 0.76 | 0.58 | 0.52 | 0.92 | 0.94 |
| 7 | Accuracy | 0.71 | 0.73 | 0.69 | 0.57 | 0.65 | 0.65 | 0.61 | 0.62 | 0.74 | 0.78 |
|  | Sensitivity | 0.60 | 0.62 | 0.74 | 0.49 | 0.68 | 0.71 | 0.98 | 1.00 | 0.75 | 0.76 |
|  | Specificity | 0.83 | 0.84 | 0.66 | 0.62 | 0.63 | 0.58 | 0.04 | 0.00 | 0.72 | 0.83 |
|  | AUC | 0.78 | 0.75 | 0.75 | 0.59 | 0.65 | 0.74 | 0.56 | 0.65 | 0.84 | 0.91 |
| 8 | Accuracy | 0.71 | 0.73 | 0.74 | 0.58 | 0.63 | 0.69 | 0.62 | 0.63 | 0.90 | 0.85 |
|  | Sensitivity | 0.61 | 0.64 | 0.79 | 0.51 | 0.68 | 0.74 | 1.00 | 1.00 | 0.91 | 0.79 |
|  | Specificity | 0.82 | 0.83 | 0.70 | 0.62 | 0.59 | 0.64 | 0.02 | 0.03 | 0.89 | 0.94 |
|  | AUC | 0.79 | 0.78 | 0.77 | 0.59 | 0.72 | 0.75 | 0.56 | 0.69 | 0.97 | 0.96 |
| 9 | Accuracy | 0.73 | 0.73 | 0.68 | 0.54 | 0.62 | 0.69 | 0.62 | 0.62 | 0.85 | 0.84 |
|  | Sensitivity | 0.61 | 0.62 | 0.68 | 0.46 | 0.68 | 0.69 | 0.98 | 0.98 | 0.86 | 0.83 |
|  | Specificity | 0.84 | 0.84 | 0.68 | 0.59 | 0.57 | 0.69 | 0.05 | 0.03 | 0.83 | 0.86 |
|  | AUC | 0.77 | 0.76 | 0.74 | 0.59 | 0.66 | 0.79 | 0.58 | 0.68 | 0.93 | 0.96 |
| 10 | Accuracy | 0.72 | 0.73 | 0.69 | 0.56 | 0.64 | 0.70 | 0.61 | 0.62 | 0.75 | 0.81 |
|  | Sensitivity | 0.60 | 0.62 | 0.75 | 0.49 | 0.68 | 0.74 | 1.00 | 1.00 | 0.77 | 0.78 |
|  | Specificity | 0.84 | 0.84 | 0.64 | 0.60 | 0.61 | 0.67 | 0.00 | 0.00 | 0.72 | 0.86 |
|  | AUC | 0.77 | 0.73 | 0.76 | 0.59 | 0.67 | 0.74 | 0.55 | 0.59 | 0.85 | 0.90 |
| 11 | Accuracy | 0.72 | 0.73 | 0.70 | 0.55 | 0.64 | 0.73 | 0.61 | 0.62 | 0.86 | 0.85 |
|  | Sensitivity | 0.60 | 0.62 | 0.74 | 0.46 | 0.68 | 0.77 | 0.98 | 1.00 | 0.85 | 0.83 |
|  | Specificity | 0.84 | 0.84 | 0.68 | 0.60 | 0.61 | 0.69 | 0.04 | 0.00 | 0.87 | 0.89 |
|  | AUC | 0.76 | 0.73 | 0.77 | 0.60 | 0.67 | 0.76 | 0.56 | 0.56 | 0.92 | 0.93 |
| 12 | Accuracy | 0.72 | 0.73 | 0.71 | 0.55 | 0.63 | 0.69 | 0.61 | 0.63 | 0.86 | 0.91 |
|  | Sensitivity | 0.60 | 0.62 | 0.70 | 0.49 | 0.64 | 0.77 | 0.99 | 1.00 | 0.87 | 0.86 |
|  | Specificity | 0.84 | 0.84 | 0.72 | 0.59 | 0.63 | 0.61 | 0.02 | 0.03 | 0.83 | 1.00 |
|  | AUC | 0.77 | 0.74 | 0.77 | 0.60 | 0.69 | 0.76 | 0.57 | 0.55 | 0.95 | 0.97 |
| 13 | Accuracy | 0.72 | 0.73 | 0.73 | 0.56 | 0.64 | 0.69 | 0.62 | 0.64 | 0.83 | 0.83 |
|  | Sensitivity | 0.60 | 0.62 | 0.79 | 0.43 | 0.70 | 0.71 | 1.00 | 1.00 | 0.83 | 0.83 |
|  | Specificity | 0.84 | 0.84 | 0.69 | 0.64 | 0.59 | 0.67 | 0.04 | 0.06 | 0.83 | 0.83 |
|  | AUC | 0.78 | 0.77 | 0.77 | 0.59 | 0.70 | 0.75 | 0.56 | 0.68 | 0.93 | 0.94 |
| 14 | Accuracy | 0.73 | 0.73 | 0.69 | 0.53 | 0.62 | 0.73 | 0.61 | 0.62 | 0.84 | 0.70 |
|  | Sensitivity | 0.61 | 0.64 | 0.72 | 0.51 | 0.62 | 0.83 | 1.00 | 1.00 | 0.86 | 0.64 |
|  | Specificity | 0.84 | 0.83 | 0.67 | 0.53 | 0.63 | 0.64 | 0.00 | 0.00 | 0.79 | 0.80 |
|  | AUC | 0.79 | 0.79 | 0.77 | 0.61 | 0.69 | 0.83 | 0.54 | 0.59 | 0.92 | 0.90 |
| 15 | Accuracy | 0.72 | 0.73 | 0.71 | 0.60 | 0.68 | 0.65 | 0.61 | 0.64 | 0.89 | 0.85 |
|  | Sensitivity | 0.60 | 0.62 | 0.75 | 0.54 | 0.72 | 0.71 | 0.99 | 1.00 | 0.89 | 0.83 |
|  | Specificity | 0.84 | 0.84 | 0.68 | 0.64 | 0.64 | 0.58 | 0.02 | 0.06 | 0.89 | 0.89 |
|  | AUC | 0.76 | 0.73 | 0.76 | 0.60 | 0.72 | 0.72 | 0.58 | 0.67 | 0.96 | 0.95 |
| 16 | Accuracy | 0.71 | 0.72 | 0.73 | 0.54 | 0.61 | 0.70 | 0.61 | 0.65 | 0.90 | 0.82 |
|  | Sensitivity | 0.60 | 0.62 | 0.81 | 0.46 | 0.70 | 0.77 | 0.99 | 1.00 | 0.90 | 0.81 |
|  | Specificity | 0.83 | 0.81 | 0.68 | 0.59 | 0.54 | 0.64 | 0.02 | 0.08 | 0.91 | 0.83 |
|  | AUC | 0.80 | 0.78 | 0.79 | 0.58 | 0.69 | 0.79 | 0.57 | 0.69 | 0.94 | 0.93 |
| 17 | Accuracy | 0.72 | 0.74 | 0.71 | 0.56 | 0.64 | 0.73 | 0.62 | 0.64 | 0.89 | 0.86 |
|  | Sensitivity | 0.60 | 0.64 | 0.75 | 0.43 | 0.68 | 0.80 | 1.00 | 1.00 | 0.89 | 0.84 |
|  | Specificity | 0.84 | 0.84 | 0.69 | 0.64 | 0.61 | 0.67 | 0.02 | 0.06 | 0.91 | 0.89 |
|  | AUC | 0.79 | 0.77 | 0.76 | 0.59 | 0.70 | 0.78 | 0.57 | 0.70 | 0.95 | 0.95 |
| 18 | Accuracy | 0.72 | 0.73 | 0.76 | 0.57 | 0.69 | 0.68 | 0.61 | 0.66 | 0.93 | 0.88 |
|  | Sensitivity | 0.60 | 0.62 | 0.79 | 0.51 | 0.70 | 0.80 | 0.99 | 1.00 | 0.94 | 0.88 |
|  | Specificity | 0.84 | 0.84 | 0.74 | 0.60 | 0.68 | 0.56 | 0.02 | 0.11 | 0.91 | 0.89 |
|  | AUC | 0.77 | 0.74 | 0.78 | 0.63 | 0.75 | 0.77 | 0.57 | 0.67 | 0.99 | 0.95 |
| 19 | Accuracy | 0.72 | 0.73 | 0.75 | 0.59 | 0.67 | 0.68 | 0.62 | 0.65 | 0.96 | 0.84 |
|  | Sensitivity | 0.60 | 0.62 | 0.77 | 0.54 | 0.75 | 0.77 | 1.00 | 1.00 | 0.94 | 0.84 |
|  | Specificity | 0.84 | 0.84 | 0.74 | 0.62 | 0.59 | 0.58 | 0.02 | 0.08 | 0.98 | 0.83 |
|  | AUC | 0.77 | 0.75 | 0.78 | 0.63 | 0.72 | 0.79 | 0.58 | 0.66 | 0.99 | 0.94 |
| 20 | Accuracy | 0.72 | 0.72 | 0.71 | 0.53 | 0.61 | 0.70 | 0.62 | 0.63 | 0.87 | 0.89 |
|  | Sensitivity | 0.60 | 0.62 | 0.75 | 0.49 | 0.64 | 0.80 | 1.00 | 1.00 | 0.89 | 0.86 |
|  | Specificity | 0.84 | 0.83 | 0.68 | 0.55 | 0.59 | 0.61 | 0.02 | 0.03 | 0.85 | 0.94 |
|  | AUC | 0.79 | 0.77 | 0.76 | 0.59 | 0.69 | 0.79 | 0.56 | 0.68 | 0.96 | 0.97 |
| 21 | Accuracy | 0.72 | 0.73 | 0.67 | 0.55 | 0.66 | 0.69 | 0.60 | 0.62 | 0.89 | 0.90 |
|  | Sensitivity | 0.60 | 0.62 | 0.72 | 0.49 | 0.72 | 0.74 | 0.99 | 1.00 | 0.89 | 0.90 |
|  | Specificity | 0.84 | 0.84 | 0.64 | 0.59 | 0.61 | 0.64 | 0.00 | 0.00 | 0.91 | 0.91 |
|  | AUC | 0.77 | 0.75 | 0.75 | 0.59 | 0.67 | 0.78 | 0.55 | 0.59 | 0.96 | 0.96 |
| 22 | Accuracy | 0.71 | 0.72 | 0.66 | 0.56 | 0.62 | 0.66 | 0.61 | 0.62 | 0.76 | 0.84 |
|  | Sensitivity | 0.60 | 0.62 | 0.70 | 0.46 | 0.68 | 0.69 | 1.00 | 1.00 | 0.79 | 0.86 |
|  | Specificity | 0.82 | 0.81 | 0.64 | 0.62 | 0.57 | 0.64 | 0.00 | 0.00 | 0.70 | 0.80 |
|  | AUC | 0.79 | 0.76 | 0.74 | 0.59 | 0.65 | 0.73 | 0.54 | 0.61 | 0.83 | 0.89 |
| 23 | Accuracy | 0.71 | 0.74 | 0.72 | 0.55 | 0.64 | 0.70 | 0.61 | 0.64 | 0.84 | 0.82 |
|  | Sensitivity | 0.62 | 0.66 | 0.77 | 0.46 | 0.66 | 0.80 | 0.99 | 1.00 | 0.84 | 0.83 |
|  | Specificity | 0.81 | 0.83 | 0.69 | 0.60 | 0.63 | 0.61 | 0.02 | 0.06 | 0.85 | 0.80 |
|  | AUC | 0.80 | 0.78 | 0.78 | 0.60 | 0.71 | 0.78 | 0.56 | 0.68 | 0.95 | 0.93 |
| 24 | Accuracy | 0.71 | 0.73 | 0.74 | 0.54 | 0.68 | 0.69 | 0.62 | 0.63 | 0.89 | 0.92 |
|  | Sensitivity | 0.60 | 0.62 | 0.75 | 0.46 | 0.72 | 0.83 | 0.99 | 1.00 | 0.89 | 0.93 |
|  | Specificity | 0.83 | 0.84 | 0.74 | 0.59 | 0.64 | 0.56 | 0.04 | 0.03 | 0.91 | 0.91 |
|  | AUC | 0.78 | 0.75 | 0.75 | 0.61 | 0.70 | 0.81 | 0.57 | 0.68 | 0.96 | 0.97 |
| 25 | Accuracy | 0.72 | 0.73 | 0.71 | 0.55 | 0.61 | 0.69 | 0.62 | 0.63 | 0.84 | 0.74 |
|  | Sensitivity | 0.62 | 0.62 | 0.72 | 0.51 | 0.64 | 0.71 | 0.99 | 1.00 | 0.83 | 0.67 |
|  | Specificity | 0.82 | 0.84 | 0.70 | 0.57 | 0.59 | 0.67 | 0.04 | 0.03 | 0.85 | 0.86 |
|  | AUC | 0.80 | 0.77 | 0.77 | 0.60 | 0.70 | 0.78 | 0.56 | 0.67 | 0.91 | 0.88 |
| 26 | Accuracy | 0.72 | 0.73 | 0.69 | 0.55 | 0.64 | 0.68 | 0.62 | 0.64 | 0.87 | 0.90 |
|  | Sensitivity | 0.60 | 0.62 | 0.74 | 0.46 | 0.66 | 0.69 | 0.99 | 1.00 | 0.85 | 0.90 |
|  | Specificity | 0.84 | 0.84 | 0.66 | 0.60 | 0.63 | 0.67 | 0.04 | 0.06 | 0.91 | 0.91 |
|  | AUC | 0.77 | 0.76 | 0.75 | 0.60 | 0.70 | 0.78 | 0.57 | 0.68 | 0.96 | 0.98 |
| 27 | Accuracy | 0.72 | 0.73 | 0.69 | 0.56 | 0.64 | 0.62 | 0.62 | 0.63 | 0.86 | 0.91 |
|  | Sensitivity | 0.60 | 0.62 | 0.74 | 0.46 | 0.70 | 0.69 | 1.00 | 1.00 | 0.87 | 0.93 |
|  | Specificity | 0.84 | 0.84 | 0.66 | 0.62 | 0.59 | 0.56 | 0.02 | 0.03 | 0.85 | 0.89 |
|  | AUC | 0.77 | 0.74 | 0.75 | 0.60 | 0.68 | 0.76 | 0.56 | 0.68 | 0.95 | 0.97 |
| 28 | Accuracy | 0.72 | 0.73 | 0.76 | 0.55 | 0.64 | 0.70 | 0.62 | 0.64 | 0.89 | 0.83 |
|  | Sensitivity | 0.60 | 0.62 | 0.81 | 0.43 | 0.68 | 0.80 | 1.00 | 1.00 | 0.89 | 0.83 |
|  | Specificity | 0.84 | 0.84 | 0.72 | 0.62 | 0.61 | 0.61 | 0.02 | 0.06 | 0.91 | 0.83 |
|  | AUC | 0.77 | 0.73 | 0.78 | 0.61 | 0.70 | 0.79 | 0.57 | 0.70 | 0.94 | 0.93 |
| 29 | Accuracy | 0.72 | 0.73 | 0.71 | 0.56 | 0.62 | 0.70 | 0.62 | 0.62 | 0.79 | 0.76 |
|  | Sensitivity | 0.60 | 0.62 | 0.74 | 0.49 | 0.64 | 0.77 | 1.00 | 1.00 | 0.79 | 0.71 |
|  | Specificity | 0.84 | 0.84 | 0.69 | 0.60 | 0.61 | 0.64 | 0.02 | 0.00 | 0.79 | 0.86 |
|  | AUC | 0.77 | 0.76 | 0.76 | 0.60 | 0.68 | 0.78 | 0.56 | 0.66 | 0.90 | 0.89 |
| 30 | Accuracy | 0.73 | 0.72 | 0.74 | 0.59 | 0.63 | 0.69 | 0.61 | 0.63 | 0.82 | 0.81 |
|  | Sensitivity | 0.62 | 0.62 | 0.79 | 0.51 | 0.66 | 0.83 | 1.00 | 1.00 | 0.83 | 0.76 |
|  | Specificity | 0.83 | 0.83 | 0.70 | 0.64 | 0.61 | 0.56 | 0.00 | 0.03 | 0.81 | 0.89 |
|  | AUC | 0.79 | 0.79 | 0.78 | 0.59 | 0.71 | 0.78 | 0.55 | 0.65 | 0.91 | 0.91 |
| *Note:* * Identifies networks with good classification performance or better in the validation (>=0.80); ~ identifies networks with fair classification performance in the validation (>=0.70). | | | | | | | | | | | |

| Supplementary Table 5. ROC diagnostics without gender for classifications using training and test data sets per network and group comparison. | | | | | | | | | | | |
| --- | --- | --- | --- | --- | --- | --- | --- | --- | --- | --- | --- |
|  | Diagnostics  (in %) | Con-Psy vs. FEP | | Con-PD vs. PDN | | Con-PD vs. PDP | | PDP vs. PDN | | Con-Psy vs. Con-PD | |
| NW |  | Training | Test | Training | Test | Training | Test | Training | Test | Training | Test |
| 1 | Accuracy | 0.74 | 0.66 | 0.62 | 0.61 | 0.69 | 0.66 | 0.66 | 0.55 | 0.88 | 0.86 |
|  | Sensitivity | 0.68 | 0.60 | 0.62 | 0.63 | 0.74 | 0.69 | 0.89 | 0.81 | 0.87 | 0.88 |
|  | Specificity | 0.81 | 0.72 | 0.62 | 0.60 | 0.64 | 0.64 | 0.30 | 0.14 | 0.89 | 0.83 |
|  | AUC | 0.83 | 0.74 | 0.72 | 0.63 | 0.75 | 0.60 | 0.63 | 0.54 | 0.96 | 0.94 |
| 2 | Accuracy | 0.75 | 0.71 | 0.65 | 0.54 | 0.68 | 0.61 | 0.64 | 0.59 | 0.93 | 0.90 |
|  | Sensitivity | 0.71 | 0.64 | 0.66 | 0.49 | 0.70 | 0.69 | 0.85 | 0.83 | 0.93 | 0.95 |
|  | Specificity | 0.80 | 0.78 | 0.64 | 0.57 | 0.66 | 0.53 | 0.30 | 0.19 | 0.92 | 0.83 |
|  | AUC | 0.84 | 0.79 | 0.72 | 0.59 | 0.76 | 0.61 | 0.64 | 0.50 | 0.99 | 0.94 |
| 3 | Accuracy | 0.77 | 0.69 | 0.65 | 0.62 | 0.70 | 0.65 | 0.68 | 0.61 | 0.86 | 0.85 |
|  | Sensitivity | 0.72 | 0.72 | 0.66 | 0.66 | 0.72 | 0.69 | 0.86 | 0.83 | 0.84 | 0.95 |
|  | Specificity | 0.81 | 0.66 | 0.64 | 0.60 | 0.68 | 0.61 | 0.39 | 0.25 | 0.91 | 0.69 |
|  | AUC | 0.85 | 0.75 | 0.71 | 0.65 | 0.80 | 0.66 | 0.65 | 0.49 | 0.94 | 0.94 |
| 4 | Accuracy | 0.74 | 0.68 | 0.65 | 0.53 | 0.68 | 0.61 | 0.66 | 0.55 | 0.93 | 0.86 |
|  | Sensitivity | 0.67 | 0.59 | 0.66 | 0.49 | 0.72 | 0.71 | 0.87 | 0.81 | 0.92 | 0.93 |
|  | Specificity | 0.82 | 0.78 | 0.64 | 0.55 | 0.64 | 0.50 | 0.34 | 0.14 | 0.94 | 0.74 |
|  | AUC | 0.83 | 0.80 | 0.72 | 0.58 | 0.75 | 0.61 | 0.63 | 0.53 | 0.98 | 0.93 |
| 5 | Accuracy | 0.73 | 0.67 | 0.64 | 0.62 | 0.67 | 0.65 | 0.64 | 0.55 | 0.89 | 0.85 |
|  | Sensitivity | 0.66 | 0.60 | 0.64 | 0.63 | 0.68 | 0.69 | 0.86 | 0.79 | 0.87 | 0.84 |
|  | Specificity | 0.81 | 0.74 | 0.63 | 0.62 | 0.66 | 0.61 | 0.30 | 0.17 | 0.91 | 0.86 |
|  | AUC | 0.83 | 0.76 | 0.72 | 0.63 | 0.75 | 0.62 | 0.63 | 0.53 | 0.96 | 0.92 |
| 6 | Accuracy | 0.73 | 0.71 | 0.62 | 0.59 | 0.70 | 0.63 | 0.63 | 0.54 | 0.86 | 0.81 |
|  | Sensitivity | 0.66 | 0.64 | 0.62 | 0.57 | 0.72 | 0.66 | 0.86 | 0.78 | 0.86 | 0.81 |
|  | Specificity | 0.80 | 0.78 | 0.62 | 0.60 | 0.68 | 0.61 | 0.27 | 0.17 | 0.85 | 0.80 |
|  | AUC | 0.82 | 0.76 | 0.72 | 0.62 | 0.74 | 0.59 | 0.63 | 0.55 | 0.93 | 0.90 |
| 7 | Accuracy | 0.74 | 0.70 | 0.64 | 0.57 | 0.69 | 0.62 | 0.59 | 0.57 | 0.78 | 0.72 |
|  | Sensitivity | 0.67 | 0.62 | 0.64 | 0.57 | 0.72 | 0.69 | 0.80 | 0.79 | 0.78 | 0.74 |
|  | Specificity | 0.81 | 0.78 | 0.63 | 0.57 | 0.66 | 0.56 | 0.27 | 0.22 | 0.77 | 0.69 |
|  | AUC | 0.83 | 0.78 | 0.72 | 0.61 | 0.73 | 0.59 | 0.64 | 0.48 | 0.87 | 0.85 |
| 8 | Accuracy | 0.74 | 0.70 | 0.65 | 0.56 | 0.71 | 0.66 | 0.66 | 0.61 | 0.91 | 0.89 |
|  | Sensitivity | 0.67 | 0.60 | 0.66 | 0.54 | 0.72 | 0.71 | 0.85 | 0.84 | 0.91 | 0.93 |
|  | Specificity | 0.81 | 0.79 | 0.64 | 0.57 | 0.70 | 0.61 | 0.36 | 0.22 | 0.92 | 0.83 |
|  | AUC | 0.84 | 0.80 | 0.72 | 0.62 | 0.77 | 0.64 | 0.65 | 0.51 | 0.98 | 0.95 |
| 9 | Accuracy | 0.75 | 0.68 | 0.64 | 0.54 | 0.71 | 0.55 | 0.69 | 0.63 | 0.87 | 0.83 |
|  | Sensitivity | 0.69 | 0.64 | 0.66 | 0.51 | 0.70 | 0.57 | 0.89 | 0.86 | 0.87 | 0.90 |
|  | Specificity | 0.81 | 0.72 | 0.63 | 0.55 | 0.71 | 0.53 | 0.38 | 0.25 | 0.87 | 0.71 |
|  | AUC | 0.84 | 0.79 | 0.72 | 0.60 | 0.76 | 0.59 | 0.65 | 0.51 | 0.96 | 0.91 |
| 10 | Accuracy | 0.75 | 0.70 | 0.64 | 0.55 | 0.68 | 0.59 | 0.62 | 0.59 | 0.76 | 0.81 |
|  | Sensitivity | 0.70 | 0.62 | 0.64 | 0.51 | 0.72 | 0.69 | 0.84 | 0.81 | 0.77 | 0.83 |
|  | Specificity | 0.81 | 0.78 | 0.63 | 0.57 | 0.64 | 0.50 | 0.27 | 0.22 | 0.74 | 0.77 |
|  | AUC | 0.82 | 0.76 | 0.72 | 0.62 | 0.74 | 0.60 | 0.64 | 0.47 | 0.86 | 0.86 |
| 11 | Accuracy | 0.75 | 0.68 | 0.66 | 0.58 | 0.72 | 0.62 | 0.64 | 0.55 | 0.86 | 0.82 |
|  | Sensitivity | 0.68 | 0.60 | 0.72 | 0.57 | 0.74 | 0.66 | 0.86 | 0.78 | 0.85 | 0.84 |
|  | Specificity | 0.82 | 0.76 | 0.62 | 0.59 | 0.70 | 0.58 | 0.30 | 0.19 | 0.87 | 0.77 |
|  | AUC | 0.82 | 0.74 | 0.72 | 0.63 | 0.75 | 0.61 | 0.64 | 0.55 | 0.94 | 0.90 |
| 12 | Accuracy | 0.74 | 0.67 | 0.64 | 0.61 | 0.68 | 0.65 | 0.66 | 0.57 | 0.87 | 0.85 |
|  | Sensitivity | 0.68 | 0.60 | 0.64 | 0.60 | 0.72 | 0.71 | 0.87 | 0.83 | 0.86 | 0.88 |
|  | Specificity | 0.81 | 0.74 | 0.63 | 0.62 | 0.64 | 0.58 | 0.32 | 0.17 | 0.89 | 0.80 |
|  | AUC | 0.83 | 0.76 | 0.73 | 0.65 | 0.76 | 0.62 | 0.64 | 0.54 | 0.96 | 0.95 |
| 13 | Accuracy | 0.75 | 0.70 | 0.67 | 0.60 | 0.72 | 0.59 | 0.66 | 0.60 | 0.86 | 0.78 |
|  | Sensitivity | 0.68 | 0.62 | 0.70 | 0.63 | 0.75 | 0.66 | 0.87 | 0.86 | 0.85 | 0.83 |
|  | Specificity | 0.82 | 0.78 | 0.66 | 0.59 | 0.70 | 0.53 | 0.34 | 0.17 | 0.87 | 0.71 |
|  | AUC | 0.83 | 0.78 | 0.72 | 0.62 | 0.77 | 0.61 | 0.64 | 0.52 | 0.94 | 0.89 |
| 14 | Accuracy | 0.76 | 0.72 | 0.65 | 0.61 | 0.70 | 0.62 | 0.67 | 0.55 | 0.86 | 0.80 |
|  | Sensitivity | 0.71 | 0.72 | 0.68 | 0.63 | 0.70 | 0.69 | 0.86 | 0.78 | 0.87 | 0.93 |
|  | Specificity | 0.81 | 0.72 | 0.63 | 0.60 | 0.70 | 0.56 | 0.38 | 0.19 | 0.85 | 0.57 |
|  | AUC | 0.84 | 0.78 | 0.71 | 0.65 | 0.79 | 0.64 | 0.64 | 0.52 | 0.94 | 0.89 |
| 15 | Accuracy | 0.74 | 0.71 | 0.64 | 0.55 | 0.72 | 0.63 | 0.66 | 0.62 | 0.90 | 0.87 |
|  | Sensitivity | 0.66 | 0.60 | 0.64 | 0.54 | 0.75 | 0.74 | 0.87 | 0.88 | 0.91 | 0.88 |
|  | Specificity | 0.83 | 0.81 | 0.63 | 0.55 | 0.68 | 0.53 | 0.32 | 0.19 | 0.89 | 0.86 |
|  | AUC | 0.82 | 0.78 | 0.72 | 0.61 | 0.76 | 0.64 | 0.64 | 0.51 | 0.96 | 0.93 |
| 16 | Accuracy | 0.77 | 0.76 | 0.64 | 0.58 | 0.68 | 0.65 | 0.68 | 0.63 | 0.89 | 0.81 |
|  | Sensitivity | 0.71 | 0.78 | 0.66 | 0.57 | 0.66 | 0.69 | 0.89 | 0.86 | 0.87 | 0.86 |
|  | Specificity | 0.83 | 0.74 | 0.63 | 0.59 | 0.70 | 0.61 | 0.36 | 0.25 | 0.92 | 0.71 |
|  | AUC | 0.85 | 0.79 | 0.73 | 0.63 | 0.77 | 0.64 | 0.64 | 0.50 | 0.96 | 0.90 |
| 17 | Accuracy | 0.75 | 0.71 | 0.64 | 0.56 | 0.71 | 0.63 | 0.67 | 0.63 | 0.88 | 0.87 |
|  | Sensitivity | 0.68 | 0.60 | 0.70 | 0.51 | 0.70 | 0.74 | 0.90 | 0.84 | 0.89 | 0.90 |
|  | Specificity | 0.82 | 0.81 | 0.61 | 0.59 | 0.71 | 0.53 | 0.32 | 0.28 | 0.87 | 0.83 |
|  | AUC | 0.83 | 0.81 | 0.72 | 0.62 | 0.76 | 0.64 | 0.65 | 0.52 | 0.96 | 0.91 |
| 18 | Accuracy | 0.73 | 0.72 | 0.69 | 0.58 | 0.72 | 0.62 | 0.62 | 0.54 | 0.89 | 0.92 |
|  | Sensitivity | 0.67 | 0.60 | 0.66 | 0.60 | 0.75 | 0.66 | 0.83 | 0.79 | 0.87 | 0.91 |
|  | Specificity | 0.80 | 0.83 | 0.70 | 0.57 | 0.70 | 0.58 | 0.30 | 0.14 | 0.91 | 0.94 |
|  | AUC | 0.83 | 0.77 | 0.74 | 0.67 | 0.80 | 0.68 | 0.62 | 0.53 | 0.97 | 0.99 |
| 19 | Accuracy | 0.75 | 0.70 | 0.72 | 0.60 | 0.72 | 0.63 | 0.62 | 0.53 | 0.92 | 0.90 |
|  | Sensitivity | 0.68 | 0.59 | 0.74 | 0.66 | 0.75 | 0.69 | 0.84 | 0.78 | 0.91 | 0.93 |
|  | Specificity | 0.82 | 0.81 | 0.71 | 0.57 | 0.68 | 0.58 | 0.29 | 0.14 | 0.94 | 0.86 |
|  | AUC | 0.83 | 0.78 | 0.75 | 0.64 | 0.79 | 0.66 | 0.62 | 0.53 | 0.97 | 0.97 |
| 20 | Accuracy | 0.75 | 0.68 | 0.65 | 0.56 | 0.70 | 0.65 | 0.64 | 0.59 | 0.93 | 0.84 |
|  | Sensitivity | 0.68 | 0.62 | 0.68 | 0.54 | 0.70 | 0.71 | 0.86 | 0.81 | 0.94 | 0.93 |
|  | Specificity | 0.82 | 0.74 | 0.63 | 0.57 | 0.70 | 0.58 | 0.30 | 0.22 | 0.91 | 0.69 |
|  | AUC | 0.83 | 0.78 | 0.72 | 0.61 | 0.77 | 0.63 | 0.63 | 0.51 | 0.98 | 0.93 |
| 21 | Accuracy | 0.73 | 0.73 | 0.65 | 0.57 | 0.68 | 0.62 | 0.65 | 0.56 | 0.91 | 0.87 |
|  | Sensitivity | 0.66 | 0.66 | 0.70 | 0.57 | 0.72 | 0.71 | 0.84 | 0.83 | 0.89 | 0.91 |
|  | Specificity | 0.81 | 0.81 | 0.62 | 0.57 | 0.64 | 0.53 | 0.36 | 0.14 | 0.94 | 0.80 |
|  | AUC | 0.83 | 0.79 | 0.72 | 0.61 | 0.76 | 0.61 | 0.64 | 0.53 | 0.97 | 0.93 |
| 22 | Accuracy | 0.74 | 0.72 | 0.63 | 0.56 | 0.69 | 0.61 | 0.62 | 0.61 | 0.78 | 0.81 |
|  | Sensitivity | 0.68 | 0.66 | 0.62 | 0.51 | 0.74 | 0.66 | 0.84 | 0.81 | 0.80 | 0.84 |
|  | Specificity | 0.80 | 0.78 | 0.63 | 0.59 | 0.64 | 0.56 | 0.29 | 0.28 | 0.74 | 0.74 |
|  | AUC | 0.83 | 0.78 | 0.72 | 0.60 | 0.74 | 0.58 | 0.64 | 0.48 | 0.83 | 0.87 |
| 23 | Accuracy | 0.78 | 0.69 | 0.66 | 0.61 | 0.69 | 0.56 | 0.66 | 0.62 | 0.87 | 0.82 |
|  | Sensitivity | 0.72 | 0.71 | 0.66 | 0.63 | 0.74 | 0.66 | 0.86 | 0.84 | 0.90 | 0.90 |
|  | Specificity | 0.84 | 0.67 | 0.66 | 0.60 | 0.64 | 0.47 | 0.34 | 0.25 | 0.83 | 0.69 |
|  | AUC | 0.85 | 0.78 | 0.73 | 0.64 | 0.78 | 0.62 | 0.65 | 0.52 | 0.96 | 0.92 |
| 24 | Accuracy | 0.75 | 0.72 | 0.65 | 0.60 | 0.66 | 0.63 | 0.64 | 0.61 | 0.89 | 0.92 |
|  | Sensitivity | 0.69 | 0.62 | 0.66 | 0.66 | 0.70 | 0.63 | 0.85 | 0.81 | 0.89 | 0.97 |
|  | Specificity | 0.82 | 0.81 | 0.64 | 0.57 | 0.63 | 0.64 | 0.32 | 0.28 | 0.89 | 0.86 |
|  | AUC | 0.83 | 0.78 | 0.72 | 0.64 | 0.77 | 0.66 | 0.64 | 0.52 | 0.97 | 0.96 |
| 25 | Accuracy | 0.73 | 0.73 | 0.64 | 0.59 | 0.71 | 0.66 | 0.65 | 0.57 | 0.82 | 0.82 |
|  | Sensitivity | 0.67 | 0.66 | 0.66 | 0.51 | 0.70 | 0.71 | 0.86 | 0.81 | 0.82 | 0.88 |
|  | Specificity | 0.80 | 0.81 | 0.62 | 0.64 | 0.71 | 0.61 | 0.32 | 0.19 | 0.83 | 0.71 |
|  | AUC | 0.84 | 0.78 | 0.72 | 0.64 | 0.78 | 0.64 | 0.63 | 0.52 | 0.91 | 0.89 |
| 26 | Accuracy | 0.75 | 0.72 | 0.66 | 0.56 | 0.68 | 0.62 | 0.64 | 0.60 | 0.91 | 0.86 |
|  | Sensitivity | 0.70 | 0.66 | 0.68 | 0.54 | 0.75 | 0.69 | 0.85 | 0.83 | 0.90 | 0.86 |
|  | Specificity | 0.81 | 0.79 | 0.64 | 0.57 | 0.61 | 0.56 | 0.32 | 0.22 | 0.92 | 0.86 |
|  | AUC | 0.83 | 0.79 | 0.72 | 0.62 | 0.78 | 0.62 | 0.64 | 0.53 | 0.98 | 0.95 |
| 27 | Accuracy | 0.77 | 0.71 | 0.66 | 0.58 | 0.66 | 0.55 | 0.66 | 0.59 | 0.89 | 0.87 |
|  | Sensitivity | 0.71 | 0.64 | 0.70 | 0.57 | 0.70 | 0.60 | 0.86 | 0.83 | 0.90 | 0.91 |
|  | Specificity | 0.82 | 0.78 | 0.64 | 0.59 | 0.63 | 0.50 | 0.36 | 0.19 | 0.89 | 0.80 |
|  | AUC | 0.84 | 0.77 | 0.72 | 0.62 | 0.75 | 0.62 | 0.64 | 0.52 | 0.97 | 0.93 |
| 28 | Accuracy | 0.76 | 0.74 | 0.69 | 0.59 | 0.71 | 0.66 | 0.66 | 0.63 | 0.87 | 0.80 |
|  | Sensitivity | 0.68 | 0.66 | 0.70 | 0.63 | 0.74 | 0.74 | 0.89 | 0.86 | 0.86 | 0.79 |
|  | Specificity | 0.84 | 0.83 | 0.69 | 0.57 | 0.68 | 0.58 | 0.32 | 0.25 | 0.89 | 0.80 |
|  | AUC | 0.82 | 0.77 | 0.73 | 0.65 | 0.78 | 0.65 | 0.65 | 0.51 | 0.96 | 0.90 |
| 29 | Accuracy | 0.76 | 0.68 | 0.64 | 0.60 | 0.71 | 0.59 | 0.69 | 0.59 | 0.79 | 0.82 |
|  | Sensitivity | 0.71 | 0.64 | 0.66 | 0.63 | 0.74 | 0.66 | 0.87 | 0.81 | 0.78 | 0.91 |
|  | Specificity | 0.81 | 0.72 | 0.63 | 0.59 | 0.68 | 0.53 | 0.39 | 0.22 | 0.81 | 0.66 |
|  | AUC | 0.83 | 0.76 | 0.72 | 0.62 | 0.76 | 0.60 | 0.64 | 0.54 | 0.91 | 0.88 |
| 30 | Accuracy | 0.76 | 0.72 | 0.65 | 0.61 | 0.70 | 0.63 | 0.65 | 0.64 | 0.85 | 0.74 |
|  | Sensitivity | 0.71 | 0.67 | 0.64 | 0.66 | 0.75 | 0.71 | 0.85 | 0.88 | 0.85 | 0.78 |
|  | Specificity | 0.81 | 0.76 | 0.66 | 0.59 | 0.64 | 0.56 | 0.34 | 0.25 | 0.85 | 0.69 |
|  | AUC | 0.84 | 0.79 | 0.73 | 0.64 | 0.78 | 0.63 | 0.64 | 0.51 | 0.91 | 0.85 |
| *Note:* * Identifies networks with good classification performance or better in the validation (>=0.80); ~ identifies networks with fair classification performance in the validation (>=0.70). | | | | | | | | | | | |

| Supplementary Table 6. ROC diagnostics without tiv in percent (%) for classifications using training and test data sets per network and group comparison. | | | | | | | | | | | |
| --- | --- | --- | --- | --- | --- | --- | --- | --- | --- | --- | --- |
|  | Diagnostics  (in %) | Con-Psy vs. FEP | | Con-PD vs. PDN | | Con-PD vs. PDP | | PDN vs. PDP | | Con-Psy vs. Con-PD | |
| NW |  | Training | Test | Training | Test | Training | Test | Training | Test | Training | Test |
| 1 | Accuracy | 0.71 | 0.72 | 0.64 | 0.63 | 0.58 | 0.49 | 0.61 | 0.64 | 0.89 | 0.88 |
|  | Sensitivity | 0.64 | 0.64 | 0.57 | 0.57 | 0.53 | 0.37 | 0.99 | 1.00 | 0.89 | 0.86 |
|  | Specificity | 0.78 | 0.81 | 0.68 | 0.67 | 0.63 | 0.61 | 0.02 | 0.06 | 0.89 | 0.91 |
|  | AUC | 0.80 | 0.80 | 0.67 | 0.64 | 0.64 | 0.55 | 0.55 | 0.59 | 0.96 | 0.94 |
| 2 | Accuracy | 0.75 | 0.73 | 0.68 | 0.62 | 0.60 | 0.49 | 0.64 | 0.63 | 0.93 | 0.89 |
|  | Sensitivity | 0.71 | 0.64 | 0.64 | 0.57 | 0.55 | 0.40 | 1.00 | 1.00 | 0.92 | 0.86 |
|  | Specificity | 0.78 | 0.83 | 0.70 | 0.66 | 0.64 | 0.58 | 0.07 | 0.03 | 0.94 | 0.94 |
|  | AUC | 0.82 | 0.80 | 0.66 | 0.65 | 0.63 | 0.56 | 0.57 | 0.64 | 0.97 | 0.97 |
| 3 | Accuracy | 0.71 | 0.75 | 0.60 | 0.62 | 0.61 | 0.52 | 0.62 | 0.63 | 0.83 | 0.88 |
|  | Sensitivity | 0.67 | 0.71 | 0.57 | 0.66 | 0.60 | 0.43 | 0.99 | 1.00 | 0.83 | 0.90 |
|  | Specificity | 0.76 | 0.79 | 0.62 | 0.60 | 0.61 | 0.61 | 0.05 | 0.03 | 0.83 | 0.86 |
|  | AUC | 0.81 | 0.81 | 0.66 | 0.64 | 0.68 | 0.61 | 0.57 | 0.65 | 0.92 | 0.95 |
| 4 | Accuracy | 0.73 | 0.72 | 0.67 | 0.62 | 0.57 | 0.52 | 0.60 | 0.63 | 0.88 | 0.85 |
|  | Sensitivity | 0.67 | 0.64 | 0.62 | 0.54 | 0.51 | 0.40 | 0.99 | 1.00 | 0.87 | 0.84 |
|  | Specificity | 0.78 | 0.81 | 0.70 | 0.67 | 0.63 | 0.64 | 0.00 | 0.03 | 0.89 | 0.86 |
|  | AUC | 0.81 | 0.81 | 0.66 | 0.65 | 0.63 | 0.53 | 0.56 | 0.59 | 0.96 | 0.96 |
| 5 | Accuracy | 0.71 | 0.72 | 0.64 | 0.63 | 0.57 | 0.48 | 0.62 | 0.63 | 0.89 | 0.86 |
|  | Sensitivity | 0.63 | 0.64 | 0.58 | 0.57 | 0.55 | 0.34 | 1.00 | 1.00 | 0.89 | 0.84 |
|  | Specificity | 0.78 | 0.81 | 0.68 | 0.67 | 0.59 | 0.61 | 0.02 | 0.03 | 0.91 | 0.89 |
|  | AUC | 0.81 | 0.81 | 0.66 | 0.65 | 0.64 | 0.55 | 0.55 | 0.60 | 0.95 | 0.95 |
| 6 | Accuracy | 0.71 | 0.74 | 0.62 | 0.62 | 0.59 | 0.49 | 0.62 | 0.63 | 0.86 | 0.81 |
|  | Sensitivity | 0.64 | 0.66 | 0.58 | 0.57 | 0.55 | 0.31 | 1.00 | 0.95 | 0.86 | 0.79 |
|  | Specificity | 0.78 | 0.83 | 0.64 | 0.66 | 0.63 | 0.67 | 0.02 | 0.11 | 0.85 | 0.83 |
|  | AUC | 0.80 | 0.80 | 0.66 | 0.64 | 0.64 | 0.49 | 0.45 | 0.57 | 0.94 | 0.91 |
| 7 | Accuracy | 0.72 | 0.73 | 0.66 | 0.61 | 0.57 | 0.48 | 0.62 | 0.63 | 0.75 | 0.77 |
|  | Sensitivity | 0.66 | 0.67 | 0.58 | 0.54 | 0.53 | 0.37 | 1.00 | 1.00 | 0.76 | 0.72 |
|  | Specificity | 0.78 | 0.79 | 0.70 | 0.66 | 0.61 | 0.58 | 0.04 | 0.03 | 0.74 | 0.86 |
|  | AUC | 0.80 | 0.80 | 0.67 | 0.65 | 0.64 | 0.55 | 0.57 | 0.61 | 0.85 | 0.91 |
| 8 | Accuracy | 0.74 | 0.72 | 0.64 | 0.57 | 0.61 | 0.48 | 0.64 | 0.63 | 0.91 | 0.86 |
|  | Sensitivity | 0.68 | 0.66 | 0.60 | 0.54 | 0.58 | 0.34 | 1.00 | 1.00 | 0.91 | 0.83 |
|  | Specificity | 0.80 | 0.79 | 0.66 | 0.59 | 0.63 | 0.61 | 0.07 | 0.03 | 0.91 | 0.91 |
|  | AUC | 0.82 | 0.80 | 0.66 | 0.63 | 0.64 | 0.59 | 0.57 | 0.65 | 0.97 | 0.95 |
| 9 | Accuracy | 0.72 | 0.74 | 0.68 | 0.57 | 0.58 | 0.54 | 0.62 | 0.62 | 0.81 | 0.85 |
|  | Sensitivity | 0.69 | 0.67 | 0.62 | 0.54 | 0.53 | 0.43 | 1.00 | 1.00 | 0.80 | 0.79 |
|  | Specificity | 0.75 | 0.81 | 0.71 | 0.59 | 0.63 | 0.64 | 0.02 | 0.00 | 0.81 | 0.94 |
|  | AUC | 0.81 | 0.79 | 0.67 | 0.64 | 0.63 | 0.55 | 0.56 | 0.63 | 0.92 | 0.94 |
| 10 | Accuracy | 0.71 | 0.73 | 0.64 | 0.61 | 0.57 | 0.46 | 0.61 | 0.62 | 0.76 | 0.77 |
|  | Sensitivity | 0.64 | 0.66 | 0.58 | 0.60 | 0.57 | 0.37 | 1.00 | 1.00 | 0.76 | 0.71 |
|  | Specificity | 0.78 | 0.81 | 0.68 | 0.62 | 0.57 | 0.56 | 0.00 | 0.00 | 0.77 | 0.89 |
|  | AUC | 0.80 | 0.80 | 0.67 | 0.63 | 0.64 | 0.55 | 0.56 | 0.61 | 0.85 | 0.91 |
| 11 | Accuracy | 0.71 | 0.75 | 0.63 | 0.60 | 0.57 | 0.48 | 0.60 | 0.62 | 0.86 | 0.84 |
|  | Sensitivity | 0.64 | 0.66 | 0.58 | 0.63 | 0.58 | 0.34 | 0.99 | 1.00 | 0.86 | 0.81 |
|  | Specificity | 0.78 | 0.84 | 0.66 | 0.59 | 0.55 | 0.61 | 0.00 | 0.00 | 0.85 | 0.89 |
|  | AUC | 0.80 | 0.80 | 0.67 | 0.63 | 0.64 | 0.53 | 0.55 | 0.61 | 0.93 | 0.93 |
| 12 | Accuracy | 0.72 | 0.73 | 0.64 | 0.62 | 0.58 | 0.52 | 0.62 | 0.62 | 0.89 | 0.87 |
|  | Sensitivity | 0.64 | 0.66 | 0.58 | 0.63 | 0.58 | 0.40 | 1.00 | 1.00 | 0.90 | 0.84 |
|  | Specificity | 0.80 | 0.81 | 0.67 | 0.62 | 0.57 | 0.64 | 0.02 | 0.00 | 0.89 | 0.91 |
|  | AUC | 0.80 | 0.81 | 0.66 | 0.64 | 0.64 | 0.46 | 0.55 | 0.60 | 0.97 | 0.95 |
| 13 | Accuracy | 0.71 | 0.76 | 0.67 | 0.60 | 0.60 | 0.52 | 0.62 | 0.63 | 0.83 | 0.85 |
|  | Sensitivity | 0.66 | 0.67 | 0.58 | 0.51 | 0.53 | 0.46 | 1.00 | 1.00 | 0.83 | 0.81 |
|  | Specificity | 0.77 | 0.84 | 0.72 | 0.66 | 0.66 | 0.58 | 0.04 | 0.03 | 0.83 | 0.91 |
|  | AUC | 0.81 | 0.82 | 0.66 | 0.64 | 0.64 | 0.55 | 0.57 | 0.64 | 0.93 | 0.94 |
| 14 | Accuracy | 0.70 | 0.76 | 0.61 | 0.60 | 0.58 | 0.49 | 0.61 | 0.63 | 0.76 | 0.84 |
|  | Sensitivity | 0.67 | 0.69 | 0.55 | 0.60 | 0.55 | 0.40 | 1.00 | 1.00 | 0.79 | 0.84 |
|  | Specificity | 0.73 | 0.83 | 0.66 | 0.60 | 0.61 | 0.58 | 0.00 | 0.03 | 0.70 | 0.83 |
|  | AUC | 0.82 | 0.80 | 0.66 | 0.63 | 0.65 | 0.60 | 0.56 | 0.58 | 0.89 | 0.93 |
| 15 | Accuracy | 0.72 | 0.74 | 0.67 | 0.57 | 0.60 | 0.49 | 0.62 | 0.64 | 0.88 | 0.89 |
|  | Sensitivity | 0.66 | 0.62 | 0.60 | 0.57 | 0.53 | 0.40 | 0.98 | 1.00 | 0.87 | 0.88 |
|  | Specificity | 0.78 | 0.86 | 0.71 | 0.57 | 0.66 | 0.58 | 0.05 | 0.06 | 0.89 | 0.91 |
|  | AUC | 0.80 | 0.80 | 0.67 | 0.60 | 0.63 | 0.54 | 0.57 | 0.62 | 0.95 | 0.95 |
| 16 | Accuracy | 0.73 | 0.73 | 0.64 | 0.63 | 0.59 | 0.51 | 0.62 | 0.62 | 0.86 | 0.83 |
|  | Sensitivity | 0.66 | 0.66 | 0.57 | 0.57 | 0.55 | 0.43 | 1.00 | 1.00 | 0.87 | 0.78 |
|  | Specificity | 0.80 | 0.81 | 0.69 | 0.67 | 0.63 | 0.58 | 0.04 | 0.00 | 0.83 | 0.91 |
|  | AUC | 0.81 | 0.83 | 0.66 | 0.64 | 0.64 | 0.57 | 0.57 | 0.65 | 0.95 | 0.93 |
| 17 | Accuracy | 0.71 | 0.75 | 0.64 | 0.61 | 0.60 | 0.51 | 0.63 | 0.63 | 0.86 | 0.86 |
|  | Sensitivity | 0.63 | 0.67 | 0.58 | 0.54 | 0.55 | 0.40 | 1.00 | 1.00 | 0.87 | 0.83 |
|  | Specificity | 0.78 | 0.83 | 0.67 | 0.66 | 0.64 | 0.61 | 0.05 | 0.03 | 0.85 | 0.91 |
|  | AUC | 0.81 | 0.81 | 0.66 | 0.63 | 0.64 | 0.57 | 0.57 | 0.64 | 0.94 | 0.95 |
| 18 | Accuracy | 0.71 | 0.73 | 0.64 | 0.58 | 0.67 | 0.56 | 0.63 | 0.63 | 0.91 | 0.92 |
|  | Sensitivity | 0.64 | 0.64 | 0.62 | 0.63 | 0.72 | 0.46 | 1.00 | 1.00 | 0.90 | 0.91 |
|  | Specificity | 0.78 | 0.83 | 0.66 | 0.55 | 0.63 | 0.67 | 0.05 | 0.03 | 0.92 | 0.94 |
|  | AUC | 0.80 | 0.81 | 0.68 | 0.63 | 0.68 | 0.60 | 0.56 | 0.64 | 0.97 | 0.98 |
| 19 | Accuracy | 0.71 | 0.73 | 0.64 | 0.58 | 0.58 | 0.46 | 0.62 | 0.62 | 0.93 | 0.89 |
|  | Sensitivity | 0.63 | 0.64 | 0.60 | 0.54 | 0.55 | 0.34 | 1.00 | 1.00 | 0.92 | 0.88 |
|  | Specificity | 0.78 | 0.83 | 0.66 | 0.60 | 0.61 | 0.58 | 0.04 | 0.00 | 0.94 | 0.91 |
|  | AUC | 0.80 | 0.81 | 0.66 | 0.63 | 0.65 | 0.58 | 0.56 | 0.64 | 0.97 | 0.98 |
| 20 | Accuracy | 0.72 | 0.74 | 0.61 | 0.59 | 0.58 | 0.51 | 0.62 | 0.62 | 0.86 | 0.88 |
|  | Sensitivity | 0.68 | 0.67 | 0.57 | 0.54 | 0.55 | 0.43 | 1.00 | 1.00 | 0.86 | 0.88 |
|  | Specificity | 0.76 | 0.81 | 0.64 | 0.62 | 0.61 | 0.58 | 0.04 | 0.00 | 0.87 | 0.89 |
|  | AUC | 0.81 | 0.79 | 0.66 | 0.63 | 0.64 | 0.57 | 0.57 | 0.65 | 0.95 | 0.97 |
| 21 | Accuracy | 0.71 | 0.73 | 0.66 | 0.62 | 0.58 | 0.52 | 0.62 | 0.62 | 0.89 | 0.87 |
|  | Sensitivity | 0.66 | 0.64 | 0.58 | 0.57 | 0.51 | 0.43 | 1.00 | 1.00 | 0.89 | 0.84 |
|  | Specificity | 0.77 | 0.83 | 0.71 | 0.66 | 0.64 | 0.61 | 0.02 | 0.00 | 0.89 | 0.91 |
|  | AUC | 0.80 | 0.81 | 0.66 | 0.65 | 0.64 | 0.52 | 0.56 | 0.62 | 0.95 | 0.97 |
| 22 | Accuracy | 0.71 | 0.76 | 0.66 | 0.62 | 0.61 | 0.56 | 0.62 | 0.63 | 0.76 | 0.77 |
|  | Sensitivity | 0.66 | 0.71 | 0.58 | 0.57 | 0.53 | 0.49 | 1.00 | 1.00 | 0.83 | 0.76 |
|  | Specificity | 0.76 | 0.81 | 0.71 | 0.66 | 0.68 | 0.64 | 0.02 | 0.03 | 0.66 | 0.80 |
|  | AUC | 0.81 | 0.82 | 0.66 | 0.65 | 0.63 | 0.55 | 0.56 | 0.61 | 0.84 | 0.87 |
| 23 | Accuracy | 0.71 | 0.76 | 0.64 | 0.62 | 0.60 | 0.51 | 0.62 | 0.63 | 0.84 | 0.83 |
|  | Sensitivity | 0.67 | 0.71 | 0.58 | 0.57 | 0.55 | 0.40 | 1.00 | 1.00 | 0.86 | 0.79 |
|  | Specificity | 0.76 | 0.81 | 0.67 | 0.66 | 0.64 | 0.61 | 0.02 | 0.03 | 0.81 | 0.89 |
|  | AUC | 0.81 | 0.82 | 0.66 | 0.64 | 0.64 | 0.57 | 0.56 | 0.61 | 0.94 | 0.95 |
| 24 | Accuracy | 0.73 | 0.74 | 0.63 | 0.61 | 0.66 | 0.51 | 0.61 | 0.61 | 0.90 | 0.92 |
|  | Sensitivity | 0.67 | 0.66 | 0.57 | 0.54 | 0.62 | 0.43 | 0.98 | 0.98 | 0.91 | 0.91 |
|  | Specificity | 0.78 | 0.83 | 0.67 | 0.66 | 0.70 | 0.58 | 0.04 | 0.00 | 0.89 | 0.94 |
|  | AUC | 0.81 | 0.81 | 0.66 | 0.64 | 0.67 | 0.57 | 0.58 | 0.63 | 0.95 | 0.98 |
| 25 | Accuracy | 0.72 | 0.77 | 0.64 | 0.61 | 0.61 | 0.49 | 0.62 | 0.62 | 0.81 | 0.85 |
|  | Sensitivity | 0.66 | 0.69 | 0.58 | 0.54 | 0.55 | 0.37 | 1.00 | 1.00 | 0.82 | 0.88 |
|  | Specificity | 0.78 | 0.84 | 0.67 | 0.66 | 0.66 | 0.61 | 0.04 | 0.00 | 0.79 | 0.80 |
|  | AUC | 0.81 | 0.82 | 0.66 | 0.65 | 0.65 | 0.56 | 0.57 | 0.63 | 0.90 | 0.91 |
| 26 | Accuracy | 0.71 | 0.75 | 0.66 | 0.61 | 0.59 | 0.48 | 0.62 | 0.62 | 0.87 | 0.91 |
|  | Sensitivity | 0.68 | 0.66 | 0.58 | 0.54 | 0.55 | 0.37 | 1.00 | 1.00 | 0.85 | 0.90 |
|  | Specificity | 0.75 | 0.84 | 0.71 | 0.66 | 0.63 | 0.58 | 0.04 | 0.00 | 0.91 | 0.94 |
|  | AUC | 0.81 | 0.80 | 0.66 | 0.65 | 0.64 | 0.56 | 0.56 | 0.64 | 0.96 | 0.97 |
| 27 | Accuracy | 0.72 | 0.76 | 0.67 | 0.61 | 0.60 | 0.49 | 0.62 | 0.63 | 0.86 | 0.90 |
|  | Sensitivity | 0.68 | 0.69 | 0.58 | 0.54 | 0.53 | 0.40 | 1.00 | 1.00 | 0.87 | 0.86 |
|  | Specificity | 0.76 | 0.83 | 0.72 | 0.66 | 0.66 | 0.58 | 0.04 | 0.03 | 0.85 | 0.97 |
|  | AUC | 0.81 | 0.80 | 0.66 | 0.65 | 0.63 | 0.55 | 0.56 | 0.64 | 0.95 | 0.97 |
| 28 | Accuracy | 0.73 | 0.76 | 0.63 | 0.61 | 0.57 | 0.51 | 0.62 | 0.62 | 0.84 | 0.88 |
|  | Sensitivity | 0.67 | 0.67 | 0.57 | 0.60 | 0.55 | 0.43 | 1.00 | 1.00 | 0.84 | 0.86 |
|  | Specificity | 0.80 | 0.84 | 0.67 | 0.62 | 0.59 | 0.58 | 0.02 | 0.00 | 0.83 | 0.91 |
|  | AUC | 0.80 | 0.80 | 0.66 | 0.62 | 0.65 | 0.57 | 0.57 | 0.62 | 0.93 | 0.95 |
| 29 | Accuracy | 0.72 | 0.76 | 0.62 | 0.63 | 0.59 | 0.52 | 0.62 | 0.63 | 0.75 | 0.83 |
|  | Sensitivity | 0.67 | 0.67 | 0.57 | 0.57 | 0.53 | 0.43 | 1.00 | 1.00 | 0.76 | 0.83 |
|  | Specificity | 0.77 | 0.84 | 0.66 | 0.67 | 0.64 | 0.61 | 0.04 | 0.03 | 0.74 | 0.83 |
|  | AUC | 0.80 | 0.81 | 0.66 | 0.64 | 0.63 | 0.55 | 0.57 | 0.64 | 0.88 | 0.93 |
| 30 | Accuracy | 0.71 | 0.74 | 0.63 | 0.58 | 0.58 | 0.52 | 0.62 | 0.62 | 0.82 | 0.73 |
|  | Sensitivity | 0.64 | 0.67 | 0.57 | 0.60 | 0.53 | 0.43 | 1.00 | 1.00 | 0.82 | 0.69 |
|  | Specificity | 0.77 | 0.81 | 0.67 | 0.57 | 0.63 | 0.61 | 0.02 | 0.00 | 0.83 | 0.80 |
|  | AUC | 0.81 | 0.81 | 0.67 | 0.61 | 0.63 | 0.56 | 0.56 | 0.61 | 0.91 | 0.89 |
| Note: * Identifies networks with good classification performance or better in the validation (>=0.80); ~ identifies networks with fair classification performance in the validation (>=0.70). | | | | | | | | | | | |

| Supplementary Table 7. ROC diagnostics without scan site for classifications using training and test data sets per network and group comparison. | | | | | | | | | | | |
| --- | --- | --- | --- | --- | --- | --- | --- | --- | --- | --- | --- |
|  | Diagnostics  (in %) | Con-Psy vs. FEP | | Con-PD vs. PDN | | Con-PD vs. PDP | | PDN vs. PDP | | Con-Psy vs. Con-PD | |
| NW |  | Training | Test | Training | Test | Training | Test | Training | Test | Training | Test |
| 1 | Accuracy | 0.59 | 0.54 | 0.60 | 0.66 | 0.57 | 0.66 | 0.62 | 0.64 | 0.87 | 0.91 |
|  | Sensitivity | 0.60 | 0.53 | 0.66 | 0.57 | 0.53 | 0.71 | 1.00 | 1.00 | 0.85 | 0.95 |
|  | Specificity | 0.59 | 0.55 | 0.56 | 0.71 | 0.61 | 0.61 | 0.02 | 0.06 | 0.91 | 0.86 |
|  | AUC | 0.61 | 0.58 | 0.68 | 0.66 | 0.64 | 0.72 | 0.55 | 0.60 | 0.94 | 0.96 |
| 2 | Accuracy | 0.58 | 0.65 | 0.59 | 0.67 | 0.62 | 0.62 | 0.61 | 0.69 | 0.93 | 0.90 |
|  | Sensitivity | 0.56 | 0.67 | 0.66 | 0.57 | 0.66 | 0.66 | 0.92 | 1.00 | 0.91 | 0.97 |
|  | Specificity | 0.59 | 0.62 | 0.55 | 0.72 | 0.59 | 0.58 | 0.13 | 0.19 | 0.96 | 0.80 |
|  | AUC | 0.62 | 0.72 | 0.68 | 0.67 | 0.66 | 0.71 | 0.59 | 0.60 | 0.98 | 0.94 |
| 3 | Accuracy | 0.61 | 0.65 | 0.60 | 0.68 | 0.64 | 0.79 | 0.62 | 0.64 | 0.86 | 0.83 |
|  | Sensitivity | 0.61 | 0.66 | 0.66 | 0.63 | 0.68 | 0.89 | 0.97 | 0.98 | 0.85 | 0.83 |
|  | Specificity | 0.60 | 0.64 | 0.56 | 0.71 | 0.61 | 0.69 | 0.07 | 0.08 | 0.87 | 0.83 |
|  | AUC | 0.64 | 0.69 | 0.68 | 0.69 | 0.71 | 0.78 | 0.57 | 0.66 | 0.94 | 0.92 |
| 4 | Accuracy | 0.60 | 0.58 | 0.61 | 0.67 | 0.62 | 0.66 | 0.63 | 0.65 | 0.89 | 0.88 |
|  | Sensitivity | 0.60 | 0.57 | 0.68 | 0.57 | 0.68 | 0.74 | 0.97 | 1.00 | 0.89 | 0.95 |
|  | Specificity | 0.60 | 0.59 | 0.56 | 0.72 | 0.57 | 0.58 | 0.11 | 0.08 | 0.91 | 0.77 |
|  | AUC | 0.63 | 0.67 | 0.68 | 0.67 | 0.67 | 0.73 | 0.57 | 0.59 | 0.97 | 0.94 |
| 5 | Accuracy | 0.61 | 0.59 | 0.60 | 0.68 | 0.61 | 0.68 | 0.62 | 0.64 | 0.86 | 0.88 |
|  | Sensitivity | 0.63 | 0.59 | 0.66 | 0.60 | 0.57 | 0.71 | 0.99 | 1.00 | 0.84 | 0.93 |
|  | Specificity | 0.59 | 0.60 | 0.56 | 0.72 | 0.64 | 0.64 | 0.04 | 0.06 | 0.89 | 0.80 |
|  | AUC | 0.61 | 0.61 | 0.68 | 0.67 | 0.64 | 0.71 | 0.57 | 0.61 | 0.95 | 0.95 |
| 6 | Accuracy | 0.58 | 0.54 | 0.62 | 0.61 | 0.63 | 0.72 | 0.59 | 0.66 | 0.81 | 0.85 |
|  | Sensitivity | 0.60 | 0.57 | 0.68 | 0.51 | 0.62 | 0.74 | 0.95 | 1.00 | 0.80 | 0.86 |
|  | Specificity | 0.57 | 0.52 | 0.59 | 0.67 | 0.64 | 0.69 | 0.04 | 0.11 | 0.83 | 0.83 |
|  | AUC | 0.61 | 0.60 | 0.68 | 0.64 | 0.62 | 0.73 | 0.57 | 0.50 | 0.91 | 0.94 |
| 7 | Accuracy | 0.61 | 0.50 | 0.66 | 0.60 | 0.59 | 0.63 | 0.64 | 0.65 | 0.74 | 0.76 |
|  | Sensitivity | 0.62 | 0.50 | 0.70 | 0.51 | 0.57 | 0.66 | 1.00 | 1.00 | 0.74 | 0.74 |
|  | Specificity | 0.59 | 0.50 | 0.63 | 0.66 | 0.61 | 0.61 | 0.07 | 0.08 | 0.75 | 0.80 |
|  | AUC | 0.65 | 0.56 | 0.68 | 0.61 | 0.60 | 0.72 | 0.56 | 0.56 | 0.85 | 0.90 |
| 8 | Accuracy | 0.65 | 0.63 | 0.62 | 0.67 | 0.62 | 0.66 | 0.64 | 0.63 | 0.93 | 0.87 |
|  | Sensitivity | 0.64 | 0.64 | 0.68 | 0.57 | 0.66 | 0.74 | 0.91 | 0.93 | 0.93 | 0.90 |
|  | Specificity | 0.66 | 0.62 | 0.59 | 0.72 | 0.59 | 0.58 | 0.21 | 0.14 | 0.92 | 0.83 |
|  | AUC | 0.69 | 0.67 | 0.70 | 0.67 | 0.67 | 0.77 | 0.61 | 0.60 | 0.98 | 0.94 |
| 9 | Accuracy | 0.59 | 0.66 | 0.60 | 0.66 | 0.61 | 0.66 | 0.61 | 0.63 | 0.89 | 0.82 |
|  | Sensitivity | 0.57 | 0.62 | 0.66 | 0.54 | 0.62 | 0.66 | 0.99 | 1.00 | 0.89 | 0.88 |
|  | Specificity | 0.61 | 0.69 | 0.56 | 0.72 | 0.59 | 0.67 | 0.02 | 0.03 | 0.89 | 0.71 |
|  | AUC | 0.62 | 0.70 | 0.68 | 0.67 | 0.65 | 0.73 | 0.56 | 0.66 | 0.96 | 0.90 |
| 10 | Accuracy | 0.57 | 0.53 | 0.61 | 0.65 | 0.61 | 0.68 | 0.62 | 0.65 | 0.74 | 0.78 |
|  | Sensitivity | 0.56 | 0.50 | 0.68 | 0.54 | 0.58 | 0.71 | 0.99 | 1.00 | 0.74 | 0.79 |
|  | Specificity | 0.58 | 0.55 | 0.57 | 0.71 | 0.63 | 0.64 | 0.05 | 0.08 | 0.75 | 0.77 |
|  | AUC | 0.62 | 0.53 | 0.68 | 0.66 | 0.62 | 0.73 | 0.58 | 0.53 | 0.84 | 0.91 |
| 11 | Accuracy | 0.59 | 0.51 | 0.59 | 0.66 | 0.63 | 0.72 | 0.61 | 0.64 | 0.82 | 0.87 |
|  | Sensitivity | 0.59 | 0.48 | 0.64 | 0.57 | 0.60 | 0.74 | 0.99 | 1.00 | 0.82 | 0.90 |
|  | Specificity | 0.59 | 0.53 | 0.56 | 0.71 | 0.66 | 0.69 | 0.02 | 0.06 | 0.83 | 0.83 |
|  | AUC | 0.60 | 0.56 | 0.68 | 0.68 | 0.64 | 0.73 | 0.57 | 0.53 | 0.91 | 0.93 |
| 12 | Accuracy | 0.61 | 0.53 | 0.61 | 0.67 | 0.58 | 0.66 | 0.61 | 0.64 | 0.89 | 0.90 |
|  | Sensitivity | 0.66 | 0.57 | 0.68 | 0.63 | 0.57 | 0.77 | 0.99 | 1.00 | 0.87 | 0.95 |
|  | Specificity | 0.57 | 0.50 | 0.56 | 0.69 | 0.59 | 0.56 | 0.02 | 0.06 | 0.91 | 0.83 |
|  | AUC | 0.62 | 0.58 | 0.68 | 0.70 | 0.65 | 0.74 | 0.57 | 0.59 | 0.95 | 0.96 |
| 13 | Accuracy | 0.58 | 0.62 | 0.64 | 0.68 | 0.61 | 0.69 | 0.63 | 0.64 | 0.86 | 0.83 |
|  | Sensitivity | 0.59 | 0.64 | 0.64 | 0.57 | 0.66 | 0.77 | 0.98 | 1.00 | 0.84 | 0.88 |
|  | Specificity | 0.58 | 0.60 | 0.63 | 0.74 | 0.57 | 0.61 | 0.09 | 0.06 | 0.89 | 0.74 |
|  | AUC | 0.66 | 0.66 | 0.71 | 0.66 | 0.66 | 0.77 | 0.56 | 0.63 | 0.94 | 0.92 |
| 14 | Accuracy | 0.58 | 0.61 | 0.62 | 0.67 | 0.66 | 0.72 | 0.61 | 0.67 | 0.85 | 0.76 |
|  | Sensitivity | 0.57 | 0.66 | 0.68 | 0.60 | 0.68 | 0.74 | 0.95 | 1.00 | 0.86 | 0.79 |
|  | Specificity | 0.59 | 0.57 | 0.59 | 0.71 | 0.64 | 0.69 | 0.07 | 0.14 | 0.83 | 0.71 |
|  | AUC | 0.62 | 0.70 | 0.69 | 0.70 | 0.72 | 0.75 | 0.57 | 0.64 | 0.93 | 0.88 |
| 15 | Accuracy | 0.55 | 0.59 | 0.58 | 0.65 | 0.62 | 0.73 | 0.60 | 0.63 | 0.91 | 0.86 |
|  | Sensitivity | 0.53 | 0.59 | 0.62 | 0.54 | 0.68 | 0.80 | 0.87 | 0.95 | 0.91 | 0.90 |
|  | Specificity | 0.57 | 0.59 | 0.55 | 0.71 | 0.57 | 0.67 | 0.18 | 0.11 | 0.92 | 0.80 |
|  | AUC | 0.61 | 0.61 | 0.69 | 0.67 | 0.68 | 0.77 | 0.61 | 0.57 | 0.97 | 0.93 |
| 16 | Accuracy | 0.65 | 0.67 | 0.61 | 0.67 | 0.62 | 0.62 | 0.62 | 0.66 | 0.88 | 0.83 |
|  | Sensitivity | 0.63 | 0.64 | 0.68 | 0.60 | 0.70 | 0.74 | 0.95 | 0.98 | 0.87 | 0.90 |
|  | Specificity | 0.66 | 0.71 | 0.57 | 0.71 | 0.55 | 0.50 | 0.11 | 0.14 | 0.89 | 0.71 |
|  | AUC | 0.69 | 0.68 | 0.71 | 0.68 | 0.68 | 0.76 | 0.58 | 0.63 | 0.96 | 0.92 |
| 17 | Accuracy | 0.58 | 0.58 | 0.60 | 0.68 | 0.65 | 0.68 | 0.65 | 0.64 | 0.89 | 0.85 |
|  | Sensitivity | 0.60 | 0.60 | 0.64 | 0.60 | 0.66 | 0.74 | 0.97 | 0.97 | 0.90 | 0.86 |
|  | Specificity | 0.56 | 0.55 | 0.57 | 0.72 | 0.64 | 0.61 | 0.16 | 0.11 | 0.87 | 0.83 |
|  | AUC | 0.65 | 0.67 | 0.69 | 0.67 | 0.68 | 0.72 | 0.59 | 0.60 | 0.96 | 0.92 |
| 18 | Accuracy | 0.63 | 0.60 | 0.61 | 0.66 | 0.64 | 0.69 | 0.62 | 0.64 | 0.93 | 0.90 |
|  | Sensitivity | 0.63 | 0.62 | 0.66 | 0.57 | 0.64 | 0.77 | 0.99 | 1.00 | 0.91 | 0.93 |
|  | Specificity | 0.64 | 0.59 | 0.57 | 0.71 | 0.64 | 0.61 | 0.05 | 0.06 | 0.96 | 0.86 |
|  | AUC | 0.67 | 0.59 | 0.70 | 0.71 | 0.74 | 0.72 | 0.55 | 0.66 | 0.98 | 0.97 |
| 19 | Accuracy | 0.58 | 0.57 | 0.64 | 0.70 | 0.66 | 0.65 | 0.61 | 0.65 | 0.95 | 0.88 |
|  | Sensitivity | 0.61 | 0.55 | 0.68 | 0.57 | 0.66 | 0.69 | 0.95 | 0.98 | 0.92 | 0.95 |
|  | Specificity | 0.55 | 0.59 | 0.62 | 0.78 | 0.66 | 0.61 | 0.07 | 0.11 | 1.00 | 0.77 |
|  | AUC | 0.64 | 0.59 | 0.71 | 0.70 | 0.73 | 0.70 | 0.57 | 0.61 | 0.99 | 0.95 |
| 20 | Accuracy | 0.57 | 0.64 | 0.59 | 0.66 | 0.65 | 0.62 | 0.59 | 0.67 | 0.91 | 0.85 |
|  | Sensitivity | 0.60 | 0.69 | 0.66 | 0.57 | 0.64 | 0.69 | 0.93 | 0.98 | 0.91 | 0.90 |
|  | Specificity | 0.53 | 0.59 | 0.54 | 0.71 | 0.66 | 0.56 | 0.05 | 0.17 | 0.91 | 0.77 |
|  | AUC | 0.60 | 0.71 | 0.68 | 0.67 | 0.70 | 0.71 | 0.57 | 0.64 | 0.98 | 0.94 |
| 21 | Accuracy | 0.61 | 0.59 | 0.60 | 0.66 | 0.59 | 0.69 | 0.62 | 0.63 | 0.91 | 0.89 |
|  | Sensitivity | 0.61 | 0.53 | 0.66 | 0.57 | 0.64 | 0.74 | 1.00 | 1.00 | 0.91 | 0.93 |
|  | Specificity | 0.61 | 0.66 | 0.56 | 0.71 | 0.54 | 0.64 | 0.04 | 0.03 | 0.92 | 0.83 |
|  | AUC | 0.62 | 0.66 | 0.69 | 0.67 | 0.65 | 0.74 | 0.55 | 0.62 | 0.97 | 0.94 |
| 22 | Accuracy | 0.61 | 0.59 | 0.65 | 0.65 | 0.61 | 0.68 | 0.62 | 0.64 | 0.75 | 0.74 |
|  | Sensitivity | 0.62 | 0.59 | 0.68 | 0.51 | 0.58 | 0.69 | 0.99 | 0.98 | 0.76 | 0.78 |
|  | Specificity | 0.60 | 0.60 | 0.63 | 0.72 | 0.64 | 0.67 | 0.04 | 0.08 | 0.74 | 0.69 |
|  | AUC | 0.67 | 0.58 | 0.69 | 0.59 | 0.60 | 0.69 | 0.57 | 0.57 | 0.84 | 0.86 |
| 23 | Accuracy | 0.69 | 0.58 | 0.62 | 0.68 | 0.67 | 0.70 | 0.63 | 0.64 | 0.87 | 0.84 |
|  | Sensitivity | 0.71 | 0.53 | 0.70 | 0.54 | 0.70 | 0.74 | 0.98 | 1.00 | 0.89 | 0.90 |
|  | Specificity | 0.66 | 0.62 | 0.57 | 0.76 | 0.64 | 0.67 | 0.09 | 0.06 | 0.85 | 0.74 |
|  | AUC | 0.73 | 0.66 | 0.70 | 0.70 | 0.67 | 0.77 | 0.56 | 0.63 | 0.96 | 0.93 |
| 24 | Accuracy | 0.55 | 0.54 | 0.61 | 0.65 | 0.65 | 0.69 | 0.62 | 0.65 | 0.94 | 0.86 |
|  | Sensitivity | 0.54 | 0.57 | 0.68 | 0.57 | 0.66 | 0.74 | 0.94 | 1.00 | 0.93 | 0.95 |
|  | Specificity | 0.56 | 0.52 | 0.56 | 0.69 | 0.64 | 0.64 | 0.11 | 0.08 | 0.94 | 0.71 |
|  | AUC | 0.60 | 0.65 | 0.69 | 0.69 | 0.72 | 0.67 | 0.58 | 0.61 | 0.98 | 0.94 |
| 25 | Accuracy | 0.64 | 0.61 | 0.59 | 0.67 | 0.61 | 0.68 | 0.61 | 0.65 | 0.84 | 0.76 |
|  | Sensitivity | 0.68 | 0.60 | 0.66 | 0.57 | 0.62 | 0.77 | 0.99 | 1.00 | 0.84 | 0.79 |
|  | Specificity | 0.60 | 0.62 | 0.54 | 0.72 | 0.59 | 0.58 | 0.02 | 0.08 | 0.85 | 0.71 |
|  | AUC | 0.66 | 0.68 | 0.69 | 0.69 | 0.68 | 0.76 | 0.56 | 0.63 | 0.93 | 0.86 |
| 26 | Accuracy | 0.61 | 0.63 | 0.59 | 0.65 | 0.62 | 0.68 | 0.61 | 0.62 | 0.93 | 0.87 |
|  | Sensitivity | 0.60 | 0.62 | 0.66 | 0.57 | 0.64 | 0.71 | 1.00 | 1.00 | 0.92 | 0.90 |
|  | Specificity | 0.63 | 0.64 | 0.55 | 0.69 | 0.61 | 0.64 | 0.00 | 0.00 | 0.94 | 0.83 |
|  | AUC | 0.62 | 0.70 | 0.69 | 0.67 | 0.67 | 0.75 | 0.53 | 0.64 | 0.98 | 0.94 |
| 27 | Accuracy | 0.62 | 0.59 | 0.60 | 0.65 | 0.60 | 0.62 | 0.60 | 0.65 | 0.89 | 0.89 |
|  | Sensitivity | 0.63 | 0.64 | 0.66 | 0.54 | 0.62 | 0.66 | 0.97 | 1.00 | 0.87 | 0.93 |
|  | Specificity | 0.60 | 0.53 | 0.56 | 0.71 | 0.57 | 0.58 | 0.04 | 0.08 | 0.91 | 0.83 |
|  | AUC | 0.64 | 0.66 | 0.69 | 0.67 | 0.68 | 0.68 | 0.56 | 0.64 | 0.97 | 0.94 |
| 28 | Accuracy | 0.58 | 0.58 | 0.61 | 0.65 | 0.65 | 0.70 | 0.64 | 0.65 | 0.87 | 0.81 |
|  | Sensitivity | 0.60 | 0.57 | 0.66 | 0.49 | 0.68 | 0.77 | 0.95 | 0.98 | 0.87 | 0.86 |
|  | Specificity | 0.57 | 0.59 | 0.57 | 0.74 | 0.63 | 0.64 | 0.14 | 0.11 | 0.87 | 0.71 |
|  | AUC | 0.61 | 0.60 | 0.71 | 0.69 | 0.69 | 0.77 | 0.57 | 0.63 | 0.96 | 0.92 |
| 29 | Accuracy | 0.59 | 0.60 | 0.61 | 0.67 | 0.61 | 0.70 | 0.60 | 0.66 | 0.83 | 0.75 |
|  | Sensitivity | 0.54 | 0.66 | 0.68 | 0.57 | 0.70 | 0.69 | 0.95 | 1.00 | 0.83 | 0.76 |
|  | Specificity | 0.64 | 0.55 | 0.56 | 0.72 | 0.52 | 0.72 | 0.05 | 0.11 | 0.83 | 0.74 |
|  | AUC | 0.60 | 0.66 | 0.68 | 0.68 | 0.66 | 0.76 | 0.57 | 0.61 | 0.91 | 0.87 |
| 30 | Accuracy | 0.62 | 0.59 | 0.65 | 0.66 | 0.64 | 0.59 | 0.60 | 0.64 | 0.84 | 0.75 |
|  | Sensitivity | 0.62 | 0.50 | 0.70 | 0.54 | 0.72 | 0.77 | 0.97 | 0.98 | 0.84 | 0.81 |
|  | Specificity | 0.63 | 0.67 | 0.62 | 0.72 | 0.57 | 0.42 | 0.04 | 0.08 | 0.85 | 0.66 |
|  | AUC | 0.70 | 0.65 | 0.72 | 0.69 | 0.68 | 0.75 | 0.58 | 0.65 | 0.94 | 0.85 |
| *Note:* * Identifies networks with good classification performance or better in the validation (>=0.80); ~ identifies networks with fair classification performance in the validation (>=0.70). | | | | | | | | | | | |
