# Supplementary material for: A multicentre study on grey matter morphometric biomarkers for classifying early schizophrenia and parkinson’s disease psychosis
Source: NPJ Parkinsons Dis. 2023 Jun 8;9:87. doi: 10.1038/s41531-023-00522-z (PMC10250419; doi:10.1038/s41531-023-00522-z)
Supplement: Supplementary file 2 — Reporting Summary [file 41531_2023_522_MOESM2_ESM.pdf]

nature portfolio

Corresponding author(s): Franziska Knolle

Last updated by author(s): Mar 31, 2023

Reporting Summary

Nature Portfolio wishes to improve the reproducibility of the work that we publish. This form provides structure for consistency and transparency in reporting. For further information on Nature Portfolio policies, see our Editorial Policies and the Editorial Policy Checklist.

Statistics

For all statistical analyses, confirm that the following items are present in the figure legend, table legend, main text, or Methods section.

n/a	Confirmed	
☐	☒	The exact sample size (*n*) for each experimental group/condition, given as a discrete number and unit of measurement
☐	☒	A statement on whether measurements were taken from distinct samples or whether the same sample was measured repeatedly
☐	☒	The statistical test(s) used AND whether they are one- or two-sided<br>*Only common tests should be described solely by name; describe more complex techniques in the Methods section.*
☐	☒	A description of all covariates tested
☐	☒	A description of any assumptions or corrections, such as tests of normality and adjustment for multiple comparisons
☐	☒	A full description of the statistical parameters including central tendency (e.g. means) or other basic estimates (e.g. regression coefficient) AND variation (e.g. standard deviation) or associated estimates of uncertainty (e.g. confidence intervals)
☐	☒	For null hypothesis testing, the test statistic (e.g. *F*, *t*, *r*) with confidence intervals, effect sizes, degrees of freedom and *P* value noted<br>*Give P values as exact values whenever suitable.*
☒	☐	For Bayesian analysis, information on the choice of priors and Markov chain Monte Carlo settings
☒	☐	For hierarchical and complex designs, identification of the appropriate level for tests and full reporting of outcomes
☐	☒	Estimates of effect sizes (e.g. Cohen's *d*, Pearson's *r*), indicating how they were calculated

Our web collection on statistics for biologists contains articles on many of the points above.

Software and code

Policy information about availability of computer code

Data collection	Pre-collected data was used.
Data analysis	R-package ‘cvequality’ (https://cran.r-project.org/web/packages/cvequality/index.html) version 0.1.3<br>Multivariate Exploratory Linear Optimized Decomposition into Independent Components (MELODIC) version 6.0.<br>SPM12<br>Rstudio Version 2023.03.0+386

For manuscripts utilizing custom algorithms or software that are central to the research but not yet described in published literature, software must be made available to editors and reviewers. We strongly encourage code deposition in a community repository (e.g. GitHub). See the Nature Portfolio guidelines for submitting code & software for further information.

Data

Policy information about availability of data

All manuscripts must include a data availability statement. This statement should provide the following information, where applicable:

- Accession codes, unique identifiers, or web links for publicly available datasets
- A description of any restrictions on data availability
- For clinical datasets or third party data, please ensure that the statement adheres to our policy

All data produced in the present study are available upon reasonable request to the authors (i.e., reasons for request should be explained). Given plausible reasons there will be no restrictions on data sharing

Human research participants

Policy information about studies involving human research participants and Sex and Gender in Research.

Reporting on sex and gender	Sex and gender were no focus of our analysis. Sex was used as a covariate in the analysis to control for differences in brain sizes.
Population characteristics	age, sex and total intra-cranial volume were used as covariates
Recruitment	Pre-collected data was used.
Ethics oversight	Ethical approval was waived by the Ethical Commission Board of the Technical University Munich

Note that full information on the approval of the study protocol must also be provided in the manuscript.

Field-specific reporting

Please select the one below that is the best fit for your research. If you are not sure, read the appropriate sections before making your selection.

☒ Life sciences ☐ Behavioural & social sciences ☐ Ecological, evolutionary & environmental sciences

For a reference copy of the document with all sections, see nature.com/documents/nr-reporting-summary-flat.pdf

Life sciences study design

All studies must disclose on these points even when the disclosure is negative.

Sample size	722
Data exclusions	n/a
Replication	predictive regression analysis used training and test data sat to validate the effect
Randomization	training and test date set were computationally defined without any external parameters.
Blinding	blinding was not necessary as no intervention was included in the study

Reporting for specific materials, systems and methods

We require information from authors about some types of materials, experimental systems and methods used in many studies. Here, indicate whether each material, system or method listed is relevant to your study. If you are not sure if a list item applies to your research, read the appropriate section before selecting a response.

Materials & experimental systems

n/a	Involved in the study
☒	☐ Antibodies
☒	☐ Eukaryotic cell lines
☒	☐ Palaeontology and archaeology
☒	☐ Animals and other organisms
☒	☐ Clinical data
☒	☐ Dual use research of concern

Methods

n/a	Involved in the study
☒	☐ ChIP-seq
☒	☐ Flow cytometry
☐	☒ MRI-based neuroimaging

Magnetic resonance imaging

Experimental design

Design type	structural MRI
Design specifications	n/a
Behavioral performance measures	n/a

Acquisition

Imaging type(s)	structural MRI
Field strength	3T
Sequence & imaging parameters	MPRAGE
Area of acquisition	whole brain

Diffusion MRI ☐ Used ☒ Not used

Preprocessing

Preprocessing software	SPM12
Normalization	T1-weighted structural images were bias field corrected and segmented into grey matter, white matter, and CSF sample-specific template representative of all 722 subjects by iterative alignment of all images, non-linear registration with modulation for linear and non-linear deformations to the MNI-ICBM152 template
Normalization template	MNI-ICBM152
Noise and artifact removal	n/a
Volume censoring	n/a

Statistical modeling & inference

Model type and settings	n/a
Effect(s) tested	n/a

Specify type of analysis: ☒ Whole brain ☐ ROI-based ☐ Both

Statistic type for inference (See Eklund et al. 2016)	n/a
Correction	n/a

Models & analysis

n/a	Involved in the study
☒	☐ Functional and/or effective connectivity
☒	☐ Graph analysis
☒	☐ Multivariate modeling or predictive analysis
